# CRISPR screens and lectin microarrays identify high mannose N-glycan regulators

**DOI:** 10.1038/s41467-024-53225-1

**Published:** 2024-11-18

**Authors:** C. Kimberly Tsui, Nicholas Twells, Jenni Durieux, Emma Doan, Jacqueline Woo, Noosha Khosrojerdi, Janiya Brooks, Ayodeji Kulepa, Brant Webster, Lara K. Mahal, Andrew Dillin

**Affiliations:** 1 Department of Molecular and Cell Biology, Howard Hughes Medical Institute, University of California, Berkeley, Berkeley, CA USA; 2 Department of Chemistry, University of Alberta, Edmonton, Canada

**Keywords:** Glycobiology, Golgi

## Abstract

Glycans play critical roles in cellular signaling and function. Unlike proteins, glycan structures are not templated from genetic sequences but synthesized by the concerted activity of many genes, making them historically challenging to study. Here, we present a strategy that utilizes CRISPR screens and lectin microarrays to uncover and characterize regulators of glycosylation. We applied this approach to study the regulation of high mannose glycans – the starting structure of all asparagine(N)-linked-glycans. We used CRISPR screens to uncover the expanded network of genes controlling high mannose levels, followed by lectin microarrays to fully measure the complex effect of select regulators on glycosylation globally. Through this, we elucidated how two high mannose regulators – TM9SF3 and the CCC complex – control complex N-glycosylation via regulating Golgi morphology and function. Notably, this allows us to interrogate Golgi function in-depth and reveals that similar disruption to Golgi morphology can lead to drastically different glycosylation outcomes. Collectively, this work demonstrates a generalizable approach for systematically dissecting the regulatory network underlying glycosylation.

## Abstract (web summary)

Glycan synthesis is regulated by the concerted activity of many genes. Here, Tsui et al. present a generalizable strategy utilizing CRISPR screens and lectin microarrays to identify and characterize regulators of glycosylation.

## Introduction

All living cells and organisms are covered with glycans—complex carbohydrates linked to proteins, lipids, and RNA^1^. Glycans play critical roles in many biological processes—intracellularly, glycans are essential for protein folding and influence the stability, localization, and activity of many proteins within and outside the cell. Extracellularly, glycans on the cell surface mediate cell-cell recognition and interactions, including many immunological responses^2^. Under many acute and chronic disease states, glycosylation can become dysregulated and actively contribute to disease progression^3–6^. For example, the high mannose glycan epitope, typically found intracellularly within the ER and the Golgi, was recently identified as a stress signal for influenza virus infection, and their cell surface presentation is suggested to cause excessive tissue damage through binding innate immune lectins and overactivating the complement pathway^7,8^. However, how high mannose or other glycan motifs are regulated at the cell surface remains relatively unknown. Understanding how cell surface glycosylation is regulated has been historically challenging due to the non-templated nature of glycans. Unlike proteins, biosynthesis of glycan structures is not directly encoded in genes. Instead, glycan synthesis is controlled by an expanded network of genes that regulate biosynthetic enzyme expression and localization, glycan trafficking, organelle function, substrate availability, and carbohydrate metabolism, producing a heterogeneous collection of glycans on the cell surface^9,10^. While the biosynthetic enzymes that directly catalyze glycosidic linkages are mostly mapped out through decades of dedicated research^10,11^, the contribution of other genes remains relatively poorly understood. In addition, changes to cell states, such as activation of proteostasis stress response pathways^12^, can also drastically alter the glycan repertoire of a cell, adding to the difficulty of understanding how glycosylation is regulated. While understanding the biology underlying a specific glycan epitope remains challenging, recent advances in glycomic techniques have enabled a comprehensive survey of the glycan landscape of cells and tissues under healthy and disease states^13^. In particular, lectin microarrays, which utilize a variety of lectins and antibodies to detect specific glycan moieties, have proven to be a powerful and highly sensitive method for uncovering glycosylation differences between biological samples^14^. Lectin microarray analyzes have identified glycan changes across many diseases and have been useful for biomarker discovery in predicting disease outcome and vaccine response^7,15–18^. However, lectin microarrays alone cannot readily reveal the underlying biology that causes the glycan change in the first place. Recent advances in CRISPR screening have proven to be a powerful tool for understanding the genetics of glycosylation. Utilizing bacterial and plant toxins that bind known glycan moieties, novel genetic regulators have been identified that control the synthesis of glycoproteins and glycolipids^19–21^. Expanding on these works, we utilized the accumulated knowledge of naturally isolated lectins and their binding specificities in CRISPR screens and lectin microarrays to identify and characterize regulators of cell surface N-glycosylation. Specifically, we applied our strategy to uncover regulators of high mannose glycans—the essential intermediate structure for all N-glycans and an important glycan epitope of the innate immune response. We first used FACS- and magnetic-based cell sorting methods to conduct genome-wide and targeted screens to uncover the expanded network of genes that control cell surface levels of high mannose N-glycans. Next, we employed lectin microarrays to measure the glycan changes comprehensively to obtain mechanistic insights into how select regulators control cell surface glycosylation. Through this, we discovered how two regulators of high mannose glycosylation—a poorly characterized gene TM9SF3 and the endocytic recycling machinery CCC complex—control complex N-glycosylation and Golgi morphology and function. Specifically, we found that loss of TM9SF3 function reduces colocalization between cis-Golgi with the trans-Golgi network (TGN) and inhibits complex N-glycan formation, while disruption to the CCC complex leads to Golgi fragmentation yet mildly increases cis-Golgi and TGN colocalization, enhancing complex N-glycan production. Notably, the unbiased interrogation of Golgi function using lectin microarray revealed that Golgi morphology changes that are similar on a surface level (i.e. fragmentation) can lead to drastically different glycosylation outcomes. Together, these findings reveal cell surface high mannose N-glycosylation regulators and validate the strategy to combine CRISPR screening with lectin microarray technologies for revealing novel regulators of glycosylation.

## Results

### UPR^ER^ activation upregulates high mannose glycans on and within cells

All N-glycans begin as the 14-sugar glycan structure (Glc_3_Man_9_GlcNAc_2_), which is added onto selected asparagine residues on nascent proteins as they enter the ER for folding^22^. As these glycoproteins mature through the ER and cis-Golgi, they transition through a high mannose stage (Man_5_-Man_9_) after initial processing steps that trim off glucose residues. Typically, these high mannose structures are further processed into more complex glycans in the Golgi, such as those elongated with repeating units of Gal and GlcNAc (“poly LacNAc”) or capped with sialic acid residues, resulting in an extensive array of mature, complex N-glycans at the cell surface^22^. When cells experience stresses such as influenza viral infection, high mannose glycans can become upregulated at the cell surface and function as a stress signal that binds innate immune lectins^7^. However, it is unknown how healthy cells maintain low levels of high mannose at the surface or how these glycans become upregulated under stress conditions. Thus, we chose to focus on the regulation of high mannose glycans with our approach.

**Fig. 1:**
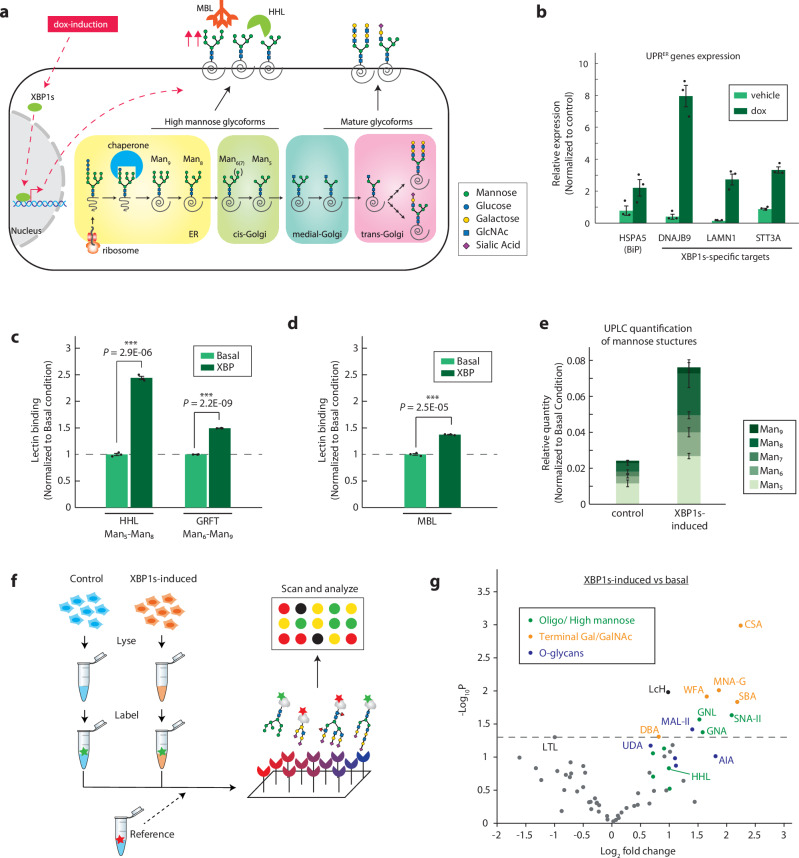
XBP1s-induction upregulates high mannose N-glycans on and within cells. **a** Schematic for dox-inducible XBP1s upregulating high mannose N-glycans. **b** RT-qPCR for targets of general UPRER and XBP1s. Gene expression is normalized to housekeeping genes GAPDH and HRPT1. **c** Fluorescent HHL and GRFT binding on A549 cells with or without dox-induction of XBP1s. Cells were treated with 2 µg/mL dox for 48 hours to overexpress XBP1s. **d** Fluorescent MBL2 binding on A549 cells with or without dox-induction of XBP1s. Cells were treated with 2 µg/mL dox for 48 hours to overexpress XBP1s. **e** UPLC quantification of high mannose N-glycan structures of A549 cells with or without XBP1s-induction. Levels of each high mannose structure are normalized to the protein amount of each replicate. **f** Schematic for lectin microarray analysis of A549s under basal or XBP1s-induced conditions. **g** Volcano plot of lectin microarray data. Median normalized log2 ratios (sample /reference) of the A549 samples are presented. Lectins are color-coded by their glycan-binding specificities. All Data are presented as mean ± s.e.m. unless otherwise indicated, and are representative of at least two independent experiments performed in triplicate with consistent results. *p* values were calculated from two-tailed Student’s *t* test.

Unfolded protein response (UPR) activation through the XBP1 pathway was required for high mannose expression in the human lung carcinoma cell line A549 in response to influenza and, in other work, was shown to induce high mannose in other cell lines in a cell-type specific manner^7,12^. We thus focused on understanding how high mannose glycans are regulated under basal and UPR^ER^-activated conditions. To this end, we built a doxycycline(dox)-inducible system to enable the overexpression of XBP1s, the key transcription factor that mediates the IRE1 branch of the UPR^ER^ response (Fig. 1a). This dox-inducible XBP1s system was lentivirally introduced into the cell line A549s. Consistent with previous work that used similar strategies to activate branches of the UPR^ER^^12,23^, our system also enabled specific upregulation of XBP1s targets (Fig. 1b).

Next, we tested whether XBP1s-induction by itself could alter cell surface high mannose glycan levels in A549. We utilized two lectins that can specifically bind high mannose N-glycan – Hippeastrum hybrid lectin (HHL), which binds N-glycan structures with Man_5_ to Man_8_^24^, and Griffithsin (GRFT), which binds Man_6_ to Man_9_^25^. We find that XBP1s activation leads to a slight but highly reproducible increase in both HHL and GRFT binding (Fig. 1c, Supplementary Fig. 1b and c). This increase in binding is reduced by cleaving off high mannose and hybrid glycans using Endoglycosidase H^3^, confirming the specificity of the lectins (Supplementary Fig. 1d). Notably, this increase in high mannose glycans on the cell surface also upregulates the binding of the complement pathway protein Mannose-binding lectin 2 (MBL2), consistent with previous reports^7^ (Fig. 1c, Supplementary Fig. 1d). In addition, partial activation of XBP1s using small molecule IXA4^26^ on wild type A549 cells also increases cell surface high mannose structures in a dose-dependent manner (Supplementary Fig. 1f). Together, these results show that activating the XBP1s branch of UPR^ER^ in A549 cells can enhance cell surface high mannose levels.

Next, to determine how each high mannose glycan structure (Man_5_ – Man_9_) changes upon XBP1s-induction on a whole cell level, we quantified all high mannose N-glycan structures using Ultra-Performance Liquid Chromatography (UPLC) with fluorescence detection^27^. Interestingly, XBP1s-induction massively upregulates all high mannose structures on a whole cell level, with the largest increase in Man 6-8 structures (Fig. 1d, Supplementary Fig. 1g). This data confirms that the high mannose expression in lung cells can be triggered by XBP1-pathway induction, providing a mechanism for glycan-based reporting of cell damage and infection to the innate immune system. Our results also suggest that the changes in cell surface glycome likely originate from changes in the early stages of N-glycosylation that occurs in the ER and Golgi.

Finally, to fully characterize the other glycosylation changes induced by XBP1s activation, we employed lectin microarray to comprehensively profile changes in glycan repertoire under basal and XBP1s-induced conditions (Fig.1e, Supplementary Data 1). We confirmed the changes in high mannose structures (HHL) and observed corresponding upregulation of oligomannose structures (Man3 to Man9) by the increased binding of lectins SNA-II, UDA, and GNA^24^ (Fig. 1f, Supplementary Fig. 1h). Interestingly, we did not observe a corresponding decrease in complex glycans but instead uncovered a concurrent upregulation of complex N-glycans capped with terminal Galactose (Gal) and N-Acetylgalactosamine (GalNAc), suggesting that cells can upregulate high mannose independently as a stress signal without compromising complex N-glycan synthesis. This is consistent with findings in influenza infection, in which high mannose upregulation did not impact the expression of most complex glycan epitopes^7^. Furthermore, we observe an upregulation of O-linked glycans (lectins: AIA, MNA, and MPL), highlighting how XBP1s induction globally alters cellular glycosylation.

### Genome-wide CRISPR screen uncovers the expanded network of genes regulating high mannose

**Fig. 2:**
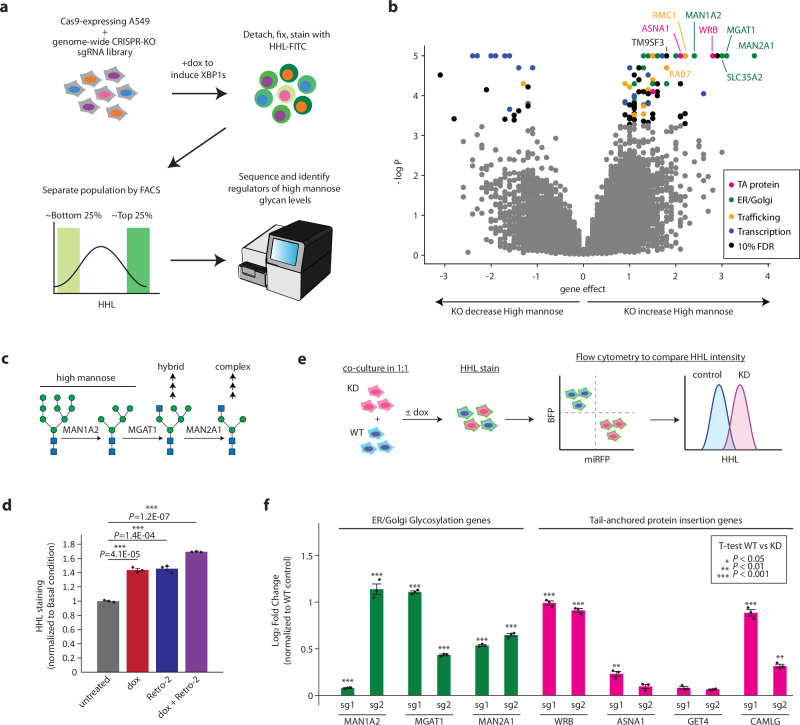
Genome-wide CRISPR screen uncovers the expanded network of genes regulating high mannose. **a** Schematic for FACS-based CRISPR screen. Cas9-expressing A549s were lentivirally transduced with a genome-wide CRISPR-deletion sgRNA library. Resulting cells were dox-treated to induce XBP1s overexpression for 48 hours. Cells were then gently lifted with Accutase, fixed, and stained with FITC-labeled HHL. The top and bottom 25% of HHL stained cells were isolated by FACS. The resulting populations were subjected to deep sequencing and analysis. The screen was performed in duplicate. **b** Volcano plot of all genes indicating effect and confidence scores for the genome-wide screen performed in duplicate. Effect and *P* values were calculated by casTLE. **c** Schematic for initial steps of N-glycan mannose-trimming and remodeling. All three enzymes indicated are hits in genome-wide screen. **d** Disruption of tail-anchored protein insertion pathway by ASNA1 inhibitor Retro-2 in wild type A549s also upregulates cell surface high mannose glycan levels. A549s were treated with treated with 2 µg/mL dox, 100 µM of Retro-2, both, or left untreated for 48 hours. Resulting cells were lifted with Accutase and stained with FITC-labeled HHL, followed by flow cytometry analysis. Data are presented as mean ± s.e.m. of median of each replicate and are representative of two independent experiments performed in triplicate with consistent results. *p* values were calculated from two-tailed Student’s *t* test. **e** Schematic for competitive binding assays for measuring changes in high mannose levels. Cells expressing sgRNAs for CRISPRi-mediated knockdown (KD) and miRFP and cells expressing a control sgRNA and BFP were cocultured in 1:1 ratio. Cells were either treated with dox to induce XBP1s or left untreated for 48 hours. Resulting cells were lifted and stained with HHL-FITC, and log2 ratio of HHL intensity of KO: control was determined using flow cytometry. **f** Validation of hits in XBP1s-induced A549s using competitive HHL binding assays. Data are presented as mean ± s.e.m. and are representative of two independent experiments performed in triplicate with consistent results. *p* values were calculated from two-tailed Student’s *t* test.

To uncover the genes beyond glycan biosynthetic enzymes controlling cell surface high mannose levels, we utilized our cellular system to conduct a genome-wide CRISPR screen (Fig. 2a). To do so, we first engineered the XBP1s-inducible A549 line to also stably express Cas9 and confirmed that concurrent expression of sgRNA targeting relevant genes, such as XBP1, can alter cell surface presentation of high mannose glycans (Supplementary Fig. 2A).

Next, we lentivirally transduced a previously validated genome-wide sgRNA knockout library^28^ into the Cas9-expressing, XBP1s-inducible A549 cells, with sgRNAs targeting all protein-coding genes with ten sgRNAs per gene and ~10,000 negative controls. The cells were then dox-treated to induce XBP1s for 48 h, fixed, and stained with FITC-labeled HHL. The population of cells with the top 25% and bottom 25% of HHL signal were selected using fluorescence-activated cell sorting (FACS), such that cells expressing sgRNAs targeting genes that suppress high mannose glycan presentation will be enriched in the top 25% and depleted in the bottom 25%, while cells expressing sgRNAs targeting genes required for high mannose glycans will be enriched in the bottom 25% and depleted in the top 25% population. The proportion of each sgRNA in the two populations was measured by deep sequencing, and significant regulators of high mannose glycan presentation were identified using casTLE^29^ (Fig. 2a).

This initial screen identified 109 known and novel regulators of high mannose glycan regulation at a 10% false discovery rate (Fig. 2b, Supplementary Data 2). Among the strongest hits were biosynthetic enzymes directly involved in N-glycan maturation – MAN1A2 and MGAT1^22^. These are Golgi-localized enzymes that act in sequential steps to remove mannoses from Man_8_ and Man_9_ structures to form Man_5_ and add a GlcNAc residue (Fig. 2c). Deletion of any of these enzymes block glycan processing to more complex structures and can therefore lead to an increase in high mannose structures. Detection of these positive controls indicates that our screening strategy worked well to identify modulators of the high mannose epitope.

Besides enzymes involved in glycosylation, our strongest hits that enhanced high mannose glycan expression were members of the tail-anchored (TA) protein insertion pathway (Fig. 2b). Knocking out four of the six canonical members (WRB, GET4, ASNA1, and CAMLG) leads to an upregulation of high mannose structures. This is likely because disruption to the TA-insertion pathway mis-localizes essential Golgi proteins^30,31^, inhibiting proper N-glycan processing in the Golgi. Indeed, when we inhibited ASNA1 using the small molecule Retro-2^31,32^, cell surface high mannose glycans became upregulated under both basal and XBP1s-induced conditions (Fig. 2d). In contrast, many of the strongest genes that caused loss of high mannose upon deletion, even in the presence of induced XBP1s, were transcriptional regulators, some of which are likely to be involved in dox-induced overexpression of XBP1s and may not be involved in direct induction of high mannose glycans by XBP1s. Given this, we decided to focus on genes whose deletion enhanced high mannose levels in our assay regardless of XBP1s.

Next, we tested whether our top hits have the same impact on high mannose glycans under basal conditions. We established individual CRISPRi-knockdown cell lines with two independent sgRNA each and assayed how the disruption of each gene impacted cell surface high mannose levels using competitive HHL binding assays (Fig. 2e). We found that all our top hits regulate high mannose levels in both basal and XBP1s-induced conditions (Fig. 2f, Supplementary Fig. 2c). Together, these results highlight the critical role of Golgi function in regulating cell surface presentation of high mannose glycans.

### Magnetic sorting-based CRISPR screens uncover additional regulators of high mannose glycans under basal and UPR^ER^-induced conditions

**Fig. 3:**
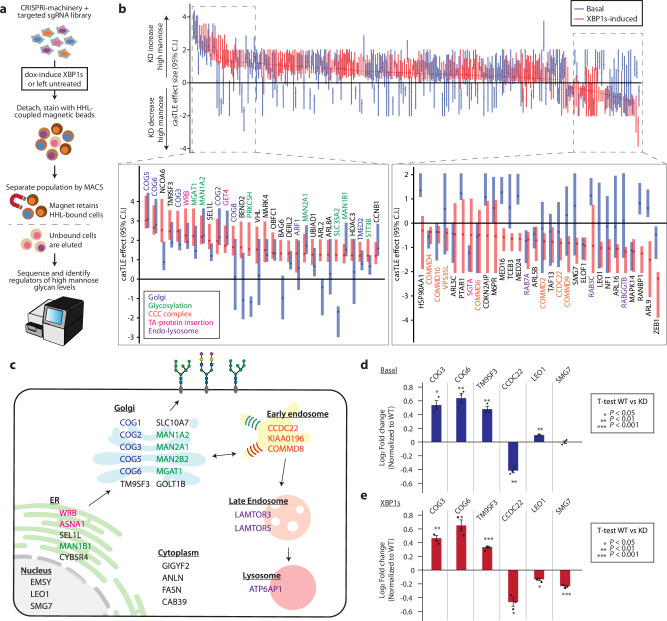
Targeted CRISPRi screen uncovers additional regulators of high mannose glycans under basal and UPR^ER^ induced conditions. **a** Schematic for MACS-based CRISPR screen. A549 cells stably expressing CRISPRi machinery and the targeted sgRNA sublibrary were either dox-treated to induce XBP1s or left untreated for 48 hours. Cells were lifted and incubated with HHL coupled to magnetic beads. The cells were then placed on a magnet in which high HHL-binding cells would be retained on the magnet, whereas the low HHL-binding cells were removed from the population. This separation was repeated twice more on each high and low HHL binding cells to improve the purity of the populations. Finally, each resulting population were subjected to deep sequencing and analysis to identify hits. The screen was performed in duplicate. **b** The maximum effect size (center value) estimated by CasTLE from both basal and XBP1s-induced conditions with five independent sgRNA per gene. The bars represent the 95% credible interval, with red representing XBP1s and blue representing basal conditions. Only genes considered to be a hit in at least one condition are shown. Genes are ordered in descending order of estimated maximum effect size of XBP1s-induced condition. The top 30 positive and negative hits are shown in the expanded panels. **c** Top 30 regulators for high mannose N-glycans with their reported subcellular localization. **d** Validation of hits in A549 under basal conditions using competitive HHL binding assays. Each gene is knocked down by co-expression of two independent sgRNAs. Data are presented as mean ± s.e.m. and are representative of two independent experiments performed in triplicate with consistent results. **e** Validation of hits in A549 under XBP1s-induced conditions using competitive HHL binding assays. Each gene is knocked down by co-expression of two independent sgRNAs. Data are presented as mean ± s.e.m. and are representative of two independent experiments performed in triplicate with consistent results. *p* values were calculated from two-tailed Student’s *t* test.

We focused our attention towards understanding high mannose regulation under basal, unstressed conditions. To do so, we generated a CRISPRi sublibrary targeting all genome-wide hits and genes functionally connected to our top hits, totaling 292 genes, with five sgRNAs each and 540 negative controls (Supplementary Data 6). Moreover, to enable faster screening at high coverage, we employed magnetic-activated cell sorting (MACS) to separate cells with high versus low levels of cell surface high mannose (Fig. 3a). We screened under both basal (untreated) or UPR^ER^ induced (XBP1s, dox) conditions to identify genes that impacted high mannose generally.

Briefly, the targeted sgRNA was lentivirally transduced into the A549 cell line with constitutively active CRISPRi machinery and dox-inducible XBP1s. The resulting cells were either treated with dox to induce XBP1s expression or left untreated. Cells were then lifted and stained with HHL conjugated to magnetic particles and separated magnetically such that cells with increased levels of high mannose on the cell surface would be retained by the magnet. In contrast, cells with less high mannose would be eluted. Each population was subjected to three rounds of separation. The proportion of each sgRNA was measured by deep sequencing and analyzed by casTLE^29^.

This strategy validated 77 hits from our genome-wide screen and further identified 111 additional genes that regulate the cell surface high mannose glycosylation under XBP1s induction. The increased number of genes identified in this secondary screen is likely due to the increased sensitivity from higher library coverage and CRISPR knockdown, which enabled essential genes to be more readily identified. Among these, 118 hits also regulate high mannose glycosylation under basal conditions. These include genes directly regulating early steps in processing the high mannose structure (e.g.MAN1A1, MAN1A2, and MGAT1). Identifying these glycosylation enzymes indicates that the screening approach worked well and has increased sensitivity in detecting high mannose regulators compared to the genome-wide screen. In addition, known Golgi regulators, such as all members of the COG complex (COG1-8), were also found to be regulators of the high mannose epitope (Fig. 3b and c, Supplementary Data 3). Top hits were validated using competitive HHL binding assays (Fig. 3d).

Our strategy also enabled us to uncover genes that, when depleted, suppress high mannose levels (Fig. 3b and c). Interestingly, these include many members of the CCC protein complex, which consists of CCDC22, CCDC93, and any of the ten COMMD proteins. The CCC complex works closely with the retriever complex to regulate protein recycling between the endosome and the cell surface^33,34^, but no glycosyltransferases have been reported as cargo. Given the tight connection between endocytic recycling and the TGN^34^, it is plausible that the CCC might be acting through the Golgi to mediate high mannose levels. However, the precise role of the CCC complex in regulating high mannose or other types of glycosylation remains unclear.

As high mannose is a key intermediate for all N-glycans, we expected that many of our hits would impact other glycan structures along the N-glycan maturation pathway. Therefore, to evaluate how the top hits effect other forms of glycosylation, we measured changes in other glycan epitopes using a panel of lectins with known specificities^24^ on live, intact cells (Supplementary Fig. 3c). We found that knocking down known Golgi regulators (COG3, COG6, and GET1) generally shifts cells to display more high and oligomannose structures and fewer branched and complex epitopes. In contrast, disrupting the CCC complex (CCDC22 and VPS35L) leads to an upregulation of mature terminal glycan epitopes such as sialic acids and GalNAc and a corresponding downregulation of high and oligomannose structures. These results show that the top hits are not merely affecting glycan density on the cell surface but impacting the cell’s glycosylation pathways.

Together, our two-tiered screening approach allowed us to uncover known and novel regulators of high mannose glycosylation beyond expected biosynthetic enzymes under both basal and XBP1s-induced conditions. However, identifying the genes alone does not provide sufficient information for understanding how these regulators, particularly ones without known connection to the biosynthetic enzymes, control glycosylation. Thus, we next sought to investigate how two regulators of opposing phenotypes—TM9SF3 and the CCC complex—act to regulate glycosylation.

### TM9SF3 regulates Golgi organization and promotes N-glycan maturation

One of the strongest hits in our screens was a poorly characterized gene TM9SF3 (Transmembrane 9 Superfamily Member 3). Knock down of this gene in A549 leads to a strong upregulation of cell surface high mannose under both basal and XBP1s-induced conditions (Fig. 3b, d, and e). Interestingly, knocking down TM9SF3 in other cell lines, K562 and Jurkat, only showed mild to no alterations to cell surface high mannose levels (Supplementary Fig. 6a and b), suggesting that TM9SF3 regulates glycosylation in a cell-type specific manner.

TM9SF3 belongs to a family of four multi-pass membrane proteins characterized by nine transmembrane domains and was previously found to be localized to the Golgi^35,36^. Moreover, another family member, TM9SF2, has been shown to regulate glycolipid synthesis^19,20^. However, the role of any TM9SFs in N-linked glycan regulation is unknown.

**Fig. 4:**
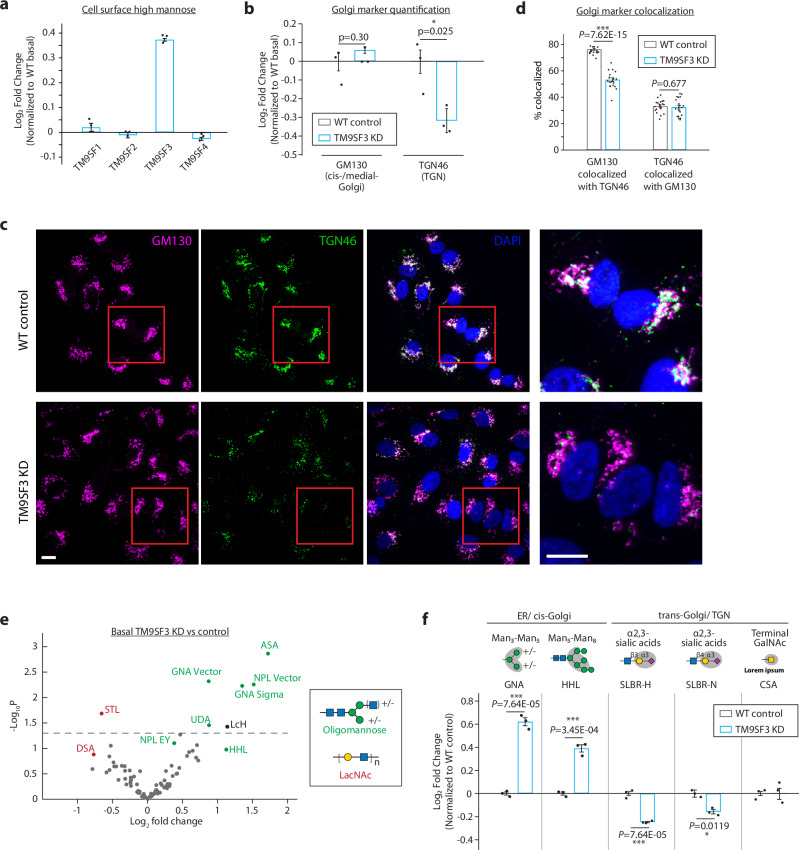
TM9SF3 regulates the Golgi organization and is required for formation of complex N-glycans. **a** Competitive HHL binding assay in A549s with each TM9SF family member knocked down. **b** Flow cytometry quantification of intracellular staining of GM130 and TGN46 in A549s. **c** Representative confocal microscopy images of TM9SF3 knockdown and wildtype control cells, co-stained with cis-/medial-Golgi marker GM130 and TGN marker TGN46. Magnified views of the red boxed areas are shown in the right-most column. Scale bars, 10 μm. Images are representative of two independent experiments performed in triplicate. **d** Percent area of each Golgi compartment co-localized with the other compartment. Colocalized area is divided by total area of the indicated Golgi marker (GM130 or TGN46) to determine the percentage of each compartment that is colocalized with the other. Data are presented as mean ± s.e.m., from at least 12 images each from wildtype or TM9SF3 knockdown of two independent experiments, with > 20 cells per image. **e** Volcano plot for lectin microarray results of A549 cells with TM9SF3 knocked down compared to wildtype control. Lectins are color-coded by their glycan-binding specificities. **f** Competitive cell surface lectin binding assay for TM9SF3 knocked down A549s compared to wild type control under basal conditions. Lectin binding specificities and location of where the modification predominately occurs are indicated. Unless otherwise indicated, all Data are presented as mean ± s.e.m. and are representative of at least three independent experiments performed in triplicate with consistent results. *p* values were calculated from two-tailed Student’s *t* test.

To begin understanding the mechanism by which TM9SF3 regulates high mannose glycosylation on the cell surface, we first validated its effect on high mannose by establishing three knockdown lines using independent sgRNAs and found that, as expected, all three lines have increased high mannose under both basal and XBP1s conditions (Supplementary Fig. 4a, b). We next we tested whether this was a general property of members of the TM9SF family. In line with our screen results, we find that only TM9SF3 acts to control high mannose levels on the cell surface (Fig. 4a, Supplementary Fig. 4c).

We reasoned that TM9SF3 might play a role in Golgi function and morphology. The Golgi has three compartments, the cis Golgi-where mannosidases reside, the medial Golgi, and TGN where complex N-glycans and sialosides are synthesized. Using intracellular staining coupled with flow cytometry quantification to study TM9SF3 knocked down cells (TM9SF3-KD), we observe a decrease in TGN marker TGN46, suggesting that there might be mild defects in TGN function (Fig. 4b, Supplementary fig. 4d). Characterization using confocal microscopy shows similar reduction in TGN46 staining (Fig. 4c). Interestingly, imaging also revealed changes in cis-and medial-Golgi morphology in TM9SF3-KD cells. Cis- and medial-Golgi compartments become highly dispersed, whereas TGN morphology and dispersion remains largely unchanged in TM9SF3-KD cells (Fig. 4c and Supplementary Fig. 4f–h). Both the dispersal of cis- and medial Golgi and reduction in TGN lead to a reduction in cis-Golgi compartments that colocalize with TGN when compared to control cells (Fig. 4d). Furthermore, using correlative light and electron microscopy (CLEM), we observe less elaborate and fewer Golgi overall when compared to wildtype control cells (Supplementary Fig. 7a and b). These results indicate that TM9SF3 regulates Golgi organization, which is essential for proper glycan maturation.

Next, we used lectin microarrays and N-glycomics to unbiasedly measure changes in global glycosylation. Because the enzymes involved in glycan processing and their Golgi localization are largely known, obtaining a comprehensive survey of the glycan repertoire can provide mechanistic insights into what glycosylation steps, and potentially Golgi compartments, are altered when our gene-of-interest is knocked down. This revealed a general upregulation of oligomannose glycans, suggesting that the early steps of N-glycan remodeling that converts high mannose into oligomannose structures, which occurs in the cis- and medial-Golgi, can proceed normally despite the altered morphology (Fig. 4e, Supplementary Fig. 4j, Supplementary Data 4). In addition, we also observed a reduction in complex LacNAc epitopes, indicating that the final steps of glycan elongation and capping needed for forming complex glycans are inhibited. Indeed, in addition to a reduction in complex LacNAc epitopes, cell surface lectin binding assays also confirm the downregulation of other complex glycan epitopes such as α2,3-sialic acids (Fig. 4f, Supplementary Fig. 4k). Similarly, N-glycomics analysis revealed that there is a shift towards high mannose glycans and a reduction in complex glycans with LacNAc and sialylated epitopes **(**Supplementary Fig. 9b and d, Supplementary Data 5**)**. Together, these results suggest that the fragmented cis- and medial-Golgi and the reduction in TGN in TM9SF3 knockdown cells may impede the trafficking of glycoproteins through Golgi compartments for glycan remodeling, resulting in a glycan repertoire enriched in high and oligomannose structures (Supplementary Fig. 10a).

### The CCC complex negatively regulates Golgi function and complex glycan formation

**Fig. 5:**
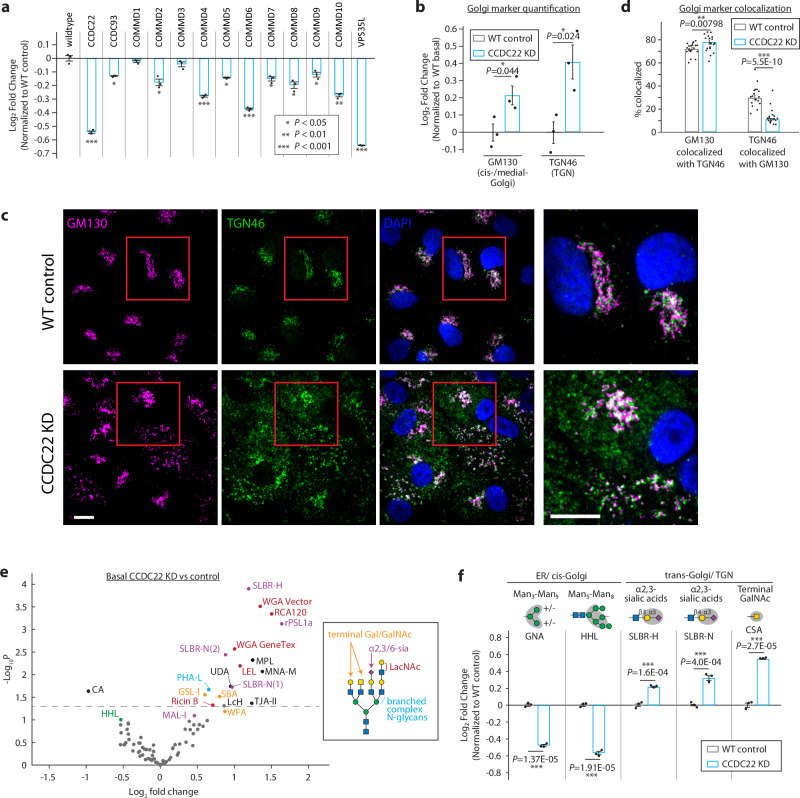
CCDC22 regulates elongation and sialylation of glycans through modulating Golgi expansion. **a** Competitive HHL binding assays on A549s for all knock down of all members of the CCC complex. Each gene is knocked down by co-expression of two independent sgRNAs. **b** Flow cytometry quantification of intracellular staining of GM130 and TGN46 in A549s in CCDC22 knockdown and wild type control cells. **c** Representative confocal microscopy images of CCDC22 knockdown and wildtype control cells, co-stained with cis-/medial-Golgi marker GM130 and TGN marker TGN46. Magnified views of the red boxed areas are shown in the right-most column. Scale bars, 10 μm. Images are representative of three independent experiments performed in triplicate. **d** Percent area of each Golgi compartment co-localized with the other compartment. Colocalized area is divided by total area of the indicated Golgi marker (GM130 or TGN46) to determine the percentage of each compartment that is colocalized with the other. Data are presented as mean ± s.e.m., from > at least 12 images each from wildtype or CCDC22 knockdown of two independent experiments, with > 20 cells per image. **e** Volcano plot for lectin microarray results of basal A549 cells with CCDC22 knocked down compared to wildtype control. Lectins are color-coded by their glycan-binding specificities. **f** Competitive cell surface lectin binding assay for CCDC22 knocked down A549s compared to wildtype control under basal conditions. Lectin binding specificities and the location of where the modification predominately occurs are indicated. Unless otherwise indicated, all Data are presented as mean ± s.e.m. and are representative of at least three independent experiments performed in triplicate with consistent results. *p* values were calculated from two-tailed Student’s *t* test.

Finally, we sought to study the role of the CCC complex in regulating N-glycosylation, given that multiple complex members are identified as regulators of the high mannose epitope. To first validate our screen results and carefully determine how each complex member impacts the high mannose epitope, we established individual knockdown lines of each member using CRISPRi with two sgRNAs each. Consistent with the screen results, we find that knocking down its core members (CCDC22 and CCDC93) and 7 of its 10 COMMD members reduces high mannose epitope on the cell surface under both basal and XBP1s-induced conditions (Fig. 5a, Supplementary Fig. 5a). Notably, knocking down VPS35L, a component that the CCC shares with the Retriever complex^33^, also down-regulates high mannose and leads to a similar glycan profile as CCDC22 knockdown (Supplementary Fig. 3c), suggesting that the CCC and Retriever complex may act together to regulate glycosylation. We also find that knocking down CCDC22 in other cell lines, K562 and Jurkat, showed similar downregulation of cell surface high mannose (Supplementary Fig. 6c and d), indicating that the CCC complex may be a common glycosylation regulator across cell types. Furthermore, the glycosylation profiles of CCC complex knockdowns are different from those of canonical Golgi regulators COG3, and COG6 knockdowns, suggesting that they play distinct roles in Golgi regulation.

Given the critical role of protein recycling in the secretory pathway, we next sought to test how the Golgi might be impacted by disruption to the CCC complex. To do so, we generated a stable A549 line with essential CCC complex component CCDC22 knocked down (Supplementary Fig. 5b). We found that CCDC22-depletion leads to a slight upregulation in both cis-/medial-Golgi marker GM130 and TGN marker TGN46 (Fig. 5b, Supplementary Fig. 5c). To characterize these Golgi changes further, we used confocal microscopy to monitor cis-/medial-Golgi as well as TGN morphology in CCDC22 knockdown cells. We observed a dispersed cis/medial-Golgi phenotype, similar to that observed in TM9SF3-KD cells despite the opposing phenotypes (Fig. 5c, Supplementary Fig. 5d and e). In the CCDC22-KD cells, we also observed a similar fragmentation and dispersion of the TGN (Fig. 5c, Supplementary Fig. 5f and g), which was consistent with previous reports^37^. Interestingly, despite the dispersion of the Golgi compartments, the cis-Golgi becomes even more colocalized with the TGN (Fig. 5c and d). We also used CLEM to assay Golgi structures but found that knocking down CCDC22 led to an accumulation of vesicle structures, some of which colocalize with TGN markers (Supplementary Fig. 8). Together, these findings led us to hypothesize that disrupting the CCC complex might either (1) trap high mannose glycans intracellularly, preventing them from reaching the cell surface, or (2) enhance high mannose remodeling into more complex glycans through increased association between the Golgi compartments.

To test these possibilities, we again utilized lectin microarray and N-glycomics to thoroughly interrogate glycosylation and Golgi function. This revealed an upregulation of complex glycan epitopes –Glycans of CCDC22 knockdown cells are more likely to be highly branched, elongated with N-acetyllactosamine (LacNAc), and capped with terminal sialic acids or galactose (Fig. 5e, Supplementary Fig. 5h, Supplementary Data 4). These changes are matched by their cell surface staining (Fig. 5f, Supplementary Fig. 5i). N-glycomics analysis also revealed an overall reduction in high mannose structures and upregulation of complex structures capped with sialic acids (Supplementary Figs. 9c and d, Supplementary Data 5). These findings strongly suggest that disruption of the CCC complex enhances the process by which high mannose glycans are remodeled into more complex N-glycans, resulting in an upregulation of complex glycans at the expense of high mannose glycans (Supplementary Fig. 10b). This may be due to the increased association between the Golgi compartments, allowing glycoproteins to be more efficiently trafficked through the Golgi and thereby promoting glycan maturation. The expanded Golgi network may also be concentrating glycan synthesis enzymes, particularly elongation and capping glycosyltransferases to generate more complex glycans. Together, our results indicate that the CCC complex is a negative regulator of Golgi function and complex N-glycan formation.

## Discussion

In this study, we present an approach utilizing CRISPR screening and lectin microarrays to identify and characterize the network of genes that regulate cell surface glycosylation. Applying this strategy, we first used genome-wide and targeted CRISPR screens to uncover regulators of high mannose glycosylation, which enabled us to identify genes beyond the known biosynthetic enzymes. We then used lectin microarrays to comprehensively measure glycosylation changes in two novel regulators – a previously poorly characterized gene TM9SF3, and the protein recycling machinery CCC complex. Our analyzes indicate that TM9SF3 is a regulator of Golgi organization and is required for proper complex N-glycan synthesis, whereas the CCC complex is revealed to be a negative regulator of Golgi function and complex glycosylation.

While it is no surprise that regulators of the Golgi would influence high mannose and other types of glycosylation, our approach allowed us to identify regulators of Golgi function in a manner traditional morphology or single glycoprotein analyzes did not provide. Notably, the use of lectin microarrays allowed us to rapidly measure changes in N- and O-glycans simultaneously, providing comprehensive insights into the state of glycosylation pathways in the cell without the need to follow specific glycosyltransferases, which can be technically challenging due to their overlapping functions as well as low protein expression. Specifically, our work found an unexpected disconnection between Golgi morphology and function, in which fragmented and dispersed Golgi appear to retain function to process glycans. Interestingly, these scattered, smaller Golgi structures observed in our TM9SF3 and CCDC22 knockdown cells are reminiscent of Golgi satellites or outposts in dendrites of neurons, where localized glycosylation events can occur in response to neuronal excitation^26,38^, suggesting that such regulation of Golgi morphology and function may be a general mechanism by which cells control glycosylation. Specifically, fragmentation and dispersion only in the cis- and medial-Golgi along with the disconnection from the TGN, like what we observe in TM9SF3-KD cells, might allow high and oligomannose glycans to bypass the intact TGN deplete cells of their complex glycan structures. On the other hand, fragmentation and rearrangement of the Golgi to bring cis- and TGN into closer contact, as we observed when the CCC complex is disrupted, might allow for concentrating specific glycosyltransferases and/or more efficient trafficking through the Golgi, enhancing remodeling and upregulating complex glycans. Follow-up studies will be required to determine whether these genes regulate glycome changes in disease states and fully elucidate how they may dynamically regulate glycosylation enzymes and glycosylation of specific proteins.

Glycosylation changes brought by changes in Golgi dynamics can have significant implications for how cells interact with the immune system. Particularly, the upregulation of complex epitopes capped with sialic acids has been shown to suppress the immune system^39^. In contrast, excess high mannose epitopes can over-activate complement pathways through interaction with mannose-binding lectin^7,8^ and promote cancer metastasis^40^. Notably, Golgi dysregulation is a feature of many diseases, including bacterial and viral pathogenic infections, cancers, and neurodegenerative diseases^41–43^. Careful research into how the Golgi alterations in various diseases regulate and change glycosylation can provide insight into how they may alter cell surface glycan signals to escape surveillance or over activate the immune system to cause chronic inflammation.

Together, our work discovered novel regulators of high mannose glycosylation and Golgi function. Additionally, our work demonstrates a readily generalizable approach combining CRISPR screens and lectin microarrays for dissecting the complex network of genes that controls the production of any glycan epitopes. This can be easily adapted to different cell types for studying the cell-type specificity of glycosylation^12,44^. Collectively, this represents a powerful method for understanding glycosylation regulation and can allow us to investigate the origins of altered glycosylation in many diseases.

## Methods

### Cell culture

A549, 293 T, Jurkat, and K562 cells were obtained from UC Berkeley Cell Culture Facility. A549s were grown in DMEM (Gibco 11966025) supplemented with 10% fetal bovine serum (FBS, Avantor, 97068-085), and 1% penicillin-streptomycin (Gibco, 15070063). 293Ts were grown in DMEM supplemented with 10% FBS, 2 mM glutamax (Gibco 35050061), and 1% penicillin-streptomycin. Jurkat and K562s were grown in RPMI (Gibco 11875093) supplemented with 10% FBS, 2 mM glutamax (Gibco 35050061), and 1% penicillin-streptomycin. All cells were cultured at 37 °C with 5% CO_2_. All knockout and knockdown cell lines were generated by transducing lentiviral constructs expressing sgRNAs into cells already with CRISPR-KO or CRISPRi machinery installed.

### RT-qPCR for UPR^ER^ targets and knockdown validations

A549 cells with dox-inducible Cas9 were either treated with 2 µg/mL doxycycline (Sigma, D3072) or equal volume of DMSO for 48 hours. Cells were washed 2x with ice cold PBS (Gibco, 10010049). TRIzol reagent (Invitrogen, 15596026) and RNeasy micro kit (Qiagen catalog no. 74004) were used in conjunction to isolate cellular RNA according to manufacture instructions. cDNA synthesis from the purified RNA was performed using QuantiTect Reverse Transcription kit (Qiagen, catalog number 205311) according to manufacturer’s instructions. SYBR Green master mix (Applied Biosystem, catalog number A46109) and primers listed in Supplementary Data 8 were used to set up qPCR reactions and analyzed on QuantStudio 6 Flex.

### Flow cytometry analysis of lectins and MBL2 binding

Cells were washed 2x with PBS and gently dissociated with 1:3 diluted Accutase (Gibco, A11105-01). Lifted cells were washed 2x with cold dPBS (HyClone, SH30264.02) and incubated with lectins at 10 µg/mL or MBL2 protein (Abcam, ab151947) at 1:50 in 3% bovine serum albumin (BSA) on ice for 1 hour. HHL was conjugated with FITC (Glycomatrix, catalog number 21511092-1), whereas His-tagged GRFT made in-house in the Mahal lab, and pre-incubated with Alexa fluor 488-labeled anti-His antibodies (Biotechne, IC0501G). After incubation with lectins or MBL2, cells were washed 2x with cold dPBS and analyzed on Attune NxT using the BL2 channel for green fluorescence. Other lectins used in flow cytometry experiments include: GNL (Vector Laboratories, FL-1241-2), Lch (EY Laboratories, F-1401-5), SNA (Bioworld 21500045-1), PNA (XXXX),CSA (EY Laboratories, BA-3201-1), PHA-L, SLBR-H, and SLBR-N (made in-house in the Mahal Lab).

### UPLC Quantification of high mannose N-glycan structures

Proteins were harvested from A549s with or without XBP1s-induction using 1% NP-40 lysis buffer -− 1% NP-40, 150 mM NaCl, and 50 mM Tris-Cl pH 8) supplemented with cOmplete protease inhibitor (Roche, 11836170001). Isolated proteins were flash frozen and sent to UC San Diego’s GlycoAnalytic Core for N-glycan analysis using Ultra-Performance Liquid Chromatography (UPLC) with fluorescent detection. Briefly, N-glycans were cleaved off by PNGase F, purified, and labeled with procainamide to allow for detection. The same amount of high mannose N-glycan structures with Man_5_-Man_9_ were spiked into each sample to allow for relative quantification of each high mannose N-glycan structure.

### Lectin microarrays

Flash frozen A549 cell pellets were washed with protease inhibitor cocktail supplemented PBS and sonicated on ice until homogenous. 20 µg of protein from each homogenized samples were then labeled with Alexa Fluor 555-NHS. A reference sample was prepared by pooling equal amounts (by total protein) of all samples and labeled with Alexa Fluor 647-NHS. Lectin microarray printing, hybridization, and data analysis was performed as previously described^14^. Details for the print are provided in the MIRAGE table (Supplementary table 1).

### FACS-based CRISPR-deletion screen for high mannose regulators

A previously established genome-wide, 10 sgRNA per gene CRISPR deletion library which is separated into 9 sublibraries was used for the genome-wide screen. A549s confirmed to stably express Cas9 and the dox-inducible XBP1s circuit was transduced with one sublibrary at a time at a multiplicity of infection (MOI) of 0.3–0.4. Cells expressing sgRNAs were selected using puromycin (Gibco, A1113803) at 1 µg/mL for 3–4 days such that > 90% of cells were mcherry positive as measured by flow cytometry. Cells were then allowed to expand for up to 7 days. Deep sequencing was used to confirm sufficient sgRNA representation in each library.

The screen was performed one sublibrary at a time due to the large amount of FACS required. For each sublibrary, cells were treated with 2 µg/mL dox for 48 hours to induce XBP1s expression. Cells were dissociated with Accutase and fixed with 4% PFA. Fixed cells were stained with HHL-FITC at 10 µg/mL in 3% BSA for 2 hours at 4 C with rotation. Cells were then washed 2x with dPBS and resuspended in 3% BSA. HHL-stained cells were sorted on BD Aria for top and bottom 25% of HHL signal, with at least 1000x coverage in each population. Cells were sorted within a week. The recovered cells were unfixed by incubating with protease K (Qiagen, 19133) overnight at 56 °C with shaking. Genomic DNA of each population was extracted using Qiagen DNA Blood Midi kit (Qiagen, 51183). The sgRNAs were amplified and prepared for sequencing with a previously described nested PCR protocol with slight modification to make sgRNA sequencing library compatible with Illumina read 1 primer. Briefly, the sgRNA-encoding constructs were first amplified with primers oKT187 and oKT188, followed by a second PCR to introduce staggered sequences and indices for multiplexing (see Supplementary Data 7 for primer sequences). The resulting PCR products were gel purified prior to sequencing on Illumina HiSeq. Hit identification was performed using CasTLE^29^. See Supplementary Data 8 for all sgRNAs used for validation.

### MACS-based CRISPR-inhibition screens

To generated CRISPRi A549s, a CRISPRi construct (pLX_311-KRAB-dCas9, gift from John Doench & William Hahn & David Root, Addgene plasmid # 96918; http://n2t.net/addgene:96918; RRID:Addgene_96918) was lentivirally introduced into the A549s expressing inducible-XBP1s. These cells were selected with blasticidin (10 µg/mL) and single cell cloned to ensure stable CRISPRi machinery expression. To generate the secondary CRISPRi screening library, sgRNAs targeting a total of 292 genes, including all genes that passed 10% FDR from the genome-wide screen as well as functionally related genes were designed using CRISPick^45,46^, along with ~500 control sgRNAs were synthesized by Twist Bioscience and cloned into pMCB320 using BstXI/BlpI overhangs after PCR amplification (see Supplementary Data 6 for complete list of genes and sgRNAs). This library was lentivirally installed into the A549s expressing CRISPRi machinery as well as inducible-XBP1s, and selected for with puromycin (1 µg/ mL).

For the screen, the 50 million library cells per condition were seeded in ten 15 cm plates. Cells were either treated with dox for 48 hours to induce XBP1s epxression or equal volume of DMSO as basal control. Cells were then lifted with Accutase, pooled, and washed 2x with dPBS. Cells were then resuspended in 10 mL 1% BSA and incubated with 100uL HHL-coupled with magnetic beads, which were prepared by mixing biotinylated HHL and MojoSort streptavidin nanobeads (BioLegend 480016) 1:1 for 30 minutes on ice. HHL-beads were allowed to bind to cells for 1 hour at 4 °C with rotation. Cells were then washed 2x with cold MojoSort buffer (BioLegend, 480017), and resuspended in MojoSort buffer. Cells were then placed on magnet and allowed to separate for 10 minutes. Unbound cells were collected into new tubes, whereas bound cells were resuspended in MojoSort buffer and allowed to separate again. After 10 minutes of separation, the unbound population is discarded, and the bound population was resuspended and allowed to separate one more time to increase purity. Similarly, the initial unbound population was placed on the magnet again, allowed to separate twice more by keeping the unbound population and discarding the bound population. Each bound and unbound populations underwent a total of three rounds of separations. A total of 10 M and 20 M cells were collected for the high mannose-low and -high populations, representing a 5000x and 10000x coverage of the sgRNA library, respectively Finally, genomic DNA were extracted from all resulting populations using Qiagen Blood Midi Prep and sgRNAs were prepared for sequencing in the same manner as the genome-wide screen. Hit identification were performed using CasTLE^29^. See Supplementary Data 8 for all sgRNAs used for validation.

### Intracellular staining and flow cytometry quantification of GM130 and TGN46

Cells were stained with standard intracellular staining techniques. Briefly, cells gently dissociated with 1:3 diluted Accutase, washed, fixed with 4% PFA (4 °C, 15 minutes), permeabilized with 0.1% Triton X-100 (room temperature, 15 minutes), and blocked with 5% FBS in dPBS (room temp, 1 hour). They were stained for 2 hours at room temp with the following primary antibodies: mouse anti-GM130 (1:250, BD Bioscience, BDB610822) and rabbit anti-TGN46 (1:500, ProteinTech, 13573-1-AP), followed by 30 minutes of incubation with the following secondary antibodies: Goat anti-mouse (1:1000, Invitrogen, A-11015), Goat anti-rabbit (1:1000, Invitrogen, A-11011). The fluorescence signal was quantified on Attune NxT.

### Immunofluorescence and confocal microscopy

Cells were grown on glass coverslips were stained using standard immunocytochemistry techniques. Briefly, cells were fixed with 4% PFA, permeabilized with 0.1% Triton X-100, blocked with 3% BSA and stained with the following antibodies: mouse anti-GM130 (1:250), rabbit anti-TGN46 (1:500), and Phalloidin Alexa Fluor 647 (Thermofisher, A30107). Cover slips were mounted using VectaShield with DAPI (Vector Laboratories, H-1800-2). All images were collected on a Nikon Ti-E inverted microscope (Nikon Instruments, Melville, NY) equipped with a Plan Apo 60× oil objective. Images were acquired using a Zyla 5.5 camera (Andor Technology), using the iQ3 acquisition software (Andor Technology).

### Correlative light and electron microscopy

Cells were grown on gridded coverslips (Mattek P35G-1.5-14-C-GRD) overnight and fixed with 4% PFA and 2% Glutaraldehyde. Cells were then permeabilized with 0.025% Triton X-100, blocked with 3% BSA and stained with mouse anti-GM130 (1:250) and rabbit anti-TGN46 (1:500). Confocal images were taken as above. Standard cell culture TEM procedure was used to prepare sample for EM imaging. Briefly, samples were embedded in resin (EMS, Hatfield) and polymerized. The glass coverslips were removed using ultra-thin Personna razor blades (EMS, Hatfield) and regions of interests identified from confocal images were excised and trimmed. The trimmed resin blocks were mounted on a large blank resin block using cyanoacrylate glue for sectioning to approximately 4-5 microns and stained with 2% uranyl acetate followed by Reynold’s lead citrate. The sections were imaged using a FEI Tecnai 12 120 kV TEM (FEI, Hillsboro) and data recorded using a Gatan Rio 16 CMOS camera with Gatan Microscopy Suite software (Gatan).

### MALDI-TOF N-glycomics analysis

For each sample, ten million cells were collected and washed twice with cold PBS. Cells were then snap frozen and sent to UC San Diego’s GlycoAnalytic Core for MALDI-TOF N-glycan analysis. Briefly, N-glycans were removed by PNGase F, purified, and permethylated. Mass spectral data were acquired using Bruker AutoFlex mass spectrometer at positive, reflectron mode. The mass spectral data were analyzed and annotated using GlycoWork Bench software and masses matched with the proposed structures were annotated. The mono-isotopic ion intensities are taken for calculation.

### Statistics and Reproducibility

All lectin staining data are presented as mean + s.e.m. and are representative of at least three independent experiments unless otherwise indicated. Two groups were compared using the two-tailed Student’s *t* test in Excel. Lectin microarray data were also compared using two-tailed Students’s t tests. Statistical analyzes for CRISPR screens were performed using casTLE. No data were excluded from analyzes.

### Reporting summary

Further information on research design is available in the Nature Portfolio Reporting Summary linked to this article.

## Supplementary information

Supplementary Information

Description of Additional Supplementary Information

Supplementary Dataset 1

Supplementary Dataset 2

Supplementary Dataset 3

Supplementary Dataset 4

Supplementary Dataset 5

Supplementary Dataset 6

Supplementary Dataset 7

Supplementary Dataset 8

Supplementary Dataset 9

Reporting Summary

## Source data

Source Data

Peer Review file

## Data Availability

The complete results of genome-wide screens and secondary screens are in Supplementary Data 2 and 3. The sgRNA counts of the screens are available through Figshare [10.6084/m9.figshare.24424777]. The complete results of lectin microarrays are in Supplementary Data 1 and 4, with raw data deposited at 10.7303/syn52845352. Data of N-glycomics is deposited on GlycoPOST^47^ at https://glycopost.glycosmos.org/entry/GPST000484. Source data are provided with this paper.

## Code availability

casTLE v.1.0 is available at https://bitbucket.org/dmorgens/castle.

## References

[1] Reily, C., Stewart, T. J., Renfrow, M. B. & Novak, J. Glycosylation in health and disease. *Nat. Rev. Nephrol.* **15**, 346–366 (2019). 30858582 10.1038/s41581-019-0129-4 PMC6590709

[2] Varki, A. Biological roles of glycans. *Glycobiology* **27**, 3–49 (2017). 27558841 10.1093/glycob/cww086 PMC5884436

[3] Ohtsubo, K. & Marth, J. D. Glycosylation in cellular mechanisms of health and disease. *Cell* **126**, 855–867 (2006). 16959566 10.1016/j.cell.2006.08.019

[4] Pinho, S. S. & Reis, C. A. Glycosylation in cancer: mechanisms and clinical implications. *Nat. Rev. Cancer* **15**, 540–555 (2015). 26289314 10.1038/nrc3982

[5] Qin, R. & Mahal, L. K. The host glycomic response to pathogens. *Curr. Opin. Struct. Biol.* **68**, 149–156 (2021). 33529786 10.1016/j.sbi.2020.12.011

[6] Ng, B. G. & Freeze, H. H. Perspectives on glycosylation and its congenital disorders. *Trends Genet* **34**, 466–476 (2018). 29606283 10.1016/j.tig.2018.03.002 PMC5959770

[7] Heindel, D. W. et al. Glycomic analysis of host response reveals high mannose as a key mediator of influenza severity. *Proc. Natl Acad. Sci. USA* **117**, 26926–26935 (2020). 33046650 10.1073/pnas.2008203117 PMC7604487

[8] Turner, M. W. The role of mannose-binding lectin in health and disease. *Mol. Immunol.* **40**, 423–429 (2003). 14568388 10.1016/s0161-5890(03)00155-x

[9] Neelamegham, S. & Mahal, L. K. Multi-level regulation of cellular glycosylation: from genes to transcript to enzyme to structure. *Curr. Opin. Struct. Biol.* **40**, 145–152 (2016). 27744149 10.1016/j.sbi.2016.09.013 PMC5161581

[10] Schjoldager, K. T., Narimatsu, Y., Joshi, H. J. & Clausen, H. Global view of human protein glycosylation pathways and functions. *Nat. Rev. Mol. Cell Biol.* **21**, 729–749 (2020). 33087899 10.1038/s41580-020-00294-x

[11] Rini, J. M., et al.) (Cold Spring Harbor Laboratory Press, 10.1101/glycobiology.4e.6 2022). 35536929

[12] Wong, M. Y. et al. XBP1s activation can globally remodel N-glycan structure distribution patterns. *Proc. Natl Acad. Sci. USA* **115**, E10089–E10098 (2018). 30305426 10.1073/pnas.1805425115 PMC6205500

[13] Chen, S., Qin, R. & Mahal, L. K. Sweet systems: technologies for glycomic analysis and their integration into systems biology. *Crit. Rev. Biochem. Mol. Biol.* **56**, 301–320 (2021). 33820453 10.1080/10409238.2021.1908953

[14] Pilobello, K. T., Slawek, D. E. & Mahal, L. K. A ratiometric lectin microarray approach to analysis of the dynamic mammalian glycome. *Proc. Natl Acad. Sci. USA* **104**, 11534–11539 (2007). 17606908 10.1073/pnas.0704954104 PMC1913879

[15] Chen, S. et al. Age-Dependent Glycomic Response to the 2009 Pandemic H1N1 Influenza Virus and Its Association with Disease Severity. *J. Proteome Res.* **19**, 4486–4495 (2020). 32981324 10.1021/acs.jproteome.0c00455 PMC7640967

[16] Heindel, D. W. et al. Glycomic analysis reveals a conserved response to bacterial sepsis induced by different bacterial pathogens. *ACS Infect. Dis.* **8**, 1075–1085 (2022). 35486714 10.1021/acsinfecdis.2c00082 PMC9112329

[17] Qin, R. et al. Prevaccination glycan markers of response to an influenza vaccine implicate the complement pathway. *J. Proteome Res.* **21**, 1974–1985 (2022). 35757850 10.1021/acs.jproteome.2c00251 PMC9361353

[18] Dang, K., Zhang, W., Jiang, S., Lin, X. & Qian, A. Application of lectin microarrays for biomarker discovery. *ChemistryOpen* **9**, 285–300 (2020). 32154049 10.1002/open.201900326 PMC7050261

[19] Tian, S. et al. Genome-wide CRISPR screens for Shiga toxins and ricin reveal Golgi proteins critical for glycosylation. *PLoS Biol.* **16**, e2006951 (2018). 30481169 10.1371/journal.pbio.2006951 PMC6258472

[20] Yamaji, T. et al. A CRISPR Screen Identifies LAPTM4A and TM9SF Proteins as Glycolipid-Regulating Factors. *iScience* **11**, 409–424 (2019). 30660999 10.1016/j.isci.2018.12.039 PMC6348303

[21] Yamaji, T. et al. A CRISPR Screen Using Subtilase Cytotoxin Identifies SLC39A9 as a Glycan-Regulating Factor. *iScience* **15**, 407–420 (2019). 31108395 10.1016/j.isci.2019.05.005 PMC6526310

[22] Stanley, P., et al.) (Cold Spring Harbor Laboratory Press, 10.1101/glycobiology.4e.9, 2022). 35536922

[23] Shoulders, M. D. et al. Stress-independent activation of XBP1s and/or ATF6 reveals three functionally diverse ER proteostasis environments. *Cell Rep.* **3**, 1279–1292 (2013). 23583182 10.1016/j.celrep.2013.03.024 PMC3754422

[24] Bojar, D. et al. A Useful Guide to Lectin Binding: Machine-Learning Directed Annotation of 57 Unique Lectin Specificities. *ACS Chem. Biol.* **17**, 2993–3012 (2022). 35084820 10.1021/acschembio.1c00689 PMC9679999

[25] Lusvarghi, S. & Bewley, C. A. Griffithsin: An Antiviral Lectin with Outstanding Therapeutic Potential. *Viruses* **8**, (2016). 10.3390/v8100296 PMC5086628 27783038

[26] Grandjean, J. M. D. et al. Pharmacologic IRE1/XBP1s activation confers targeted ER proteostasis reprogramming. *Nat. Chem. Biol.* **16**, 1052–1061 (2020). 32690944 10.1038/s41589-020-0584-z PMC7502540

[27] Klapoetke, S., Zhang, J., Becht, S., Gu, X. & Ding, X. The evaluation of a novel approach for the profiling and identification of N-linked glycan with a procainamide tag by HPLC with fluorescent and mass spectrometric detection. *J. Pharm. Biomed. Anal.* **53**, 315–324 (2010). 20418045 10.1016/j.jpba.2010.03.045

[28] Morgens, D. W. et al. Genome-scale measurement of off-target activity using Cas9 toxicity in high-throughput screens. *Nat. Commun.* **8**, 15178 (2017). 28474669 10.1038/ncomms15178 PMC5424143

[29] Morgens, D. W., Deans, R. M., Li, A. & Bassik, M. C. Systematic comparison of CRISPR/Cas9 and RNAi screens for essential genes. *Nat. Biotechnol.* **34**, 634–636 (2016). 27159373 10.1038/nbt.3567 PMC4900911

[30] Hegde, R. S. & Keenan, R. J. Tail-anchored membrane protein insertion into the endoplasmic reticulum. *Nat. Rev. Mol. Cell Biol.* **12**, 787–798 (2011). 22086371 10.1038/nrm3226 PMC3760496

[31] Morgens, D. W. et al. Retro-2 protects cells from ricin toxicity by inhibiting ASNA1-mediated ER targeting and insertion of tail-anchored proteins. *eLife* **8**, e48434 (2019). 31674906 10.7554/eLife.48434 PMC6858068

[32] Stechmann, B. et al. Inhibition of retrograde transport protects mice from lethal ricin challenge. *Cell* **141**, 231–242 (2010). 20403321 10.1016/j.cell.2010.01.043

[33] Singla, A. et al. Endosomal PI(3)P regulation by the COMMD/CCDC22/CCDC93 (CCC) complex controls membrane protein recycling. *Nat. Commun.* **10**, 4271 (2019). 31537807 10.1038/s41467-019-12221-6 PMC6753146

[34] Chen, K.-E., Healy, M. D. & Collins, B. M. Towards a molecular understanding of endosomal trafficking by Retromer and Retriever. *Traffic* **20**, 465–478 (2019). 30993794 10.1111/tra.12649

[35] Chluba-de Tapia, J., de Tapia, M., Jäggin, V. & Eberle, A. N. Cloning of a human multispanning membrane protein cDNA: evidence for a new protein family. *Gene* **197**, 195–204 (1997). 9332367 10.1016/s0378-1119(97)00263-1

[36] Au, C. E. et al. Expression, sorting, and segregation of Golgi proteins during germ cell differentiation in the testis. *Mol. Biol. Cell* **26**, 4015–4032 (2015). 25808494 10.1091/mbc.E14-12-1632 PMC4710233

[37] Phillips-Krawczak, C. A. et al. COMMD1 is linked to the WASH complex and regulates endosomal trafficking of the copper transporter ATP7A. *Mol. Biol. Cell* **26**, 91–103 (2015). 25355947 10.1091/mbc.E14-06-1073 PMC4279232

[38] Thayer, D. A., Jan, Y. N. & Jan, L. Y. Increased neuronal activity fragments the Golgi complex. *Proc. Natl Acad. Sci. USA* **110**, 1482–1487 (2013). 23297202 10.1073/pnas.1220978110 PMC3557034

[39] Varki, A. & Gagneux, P. Multifarious roles of sialic acids in immunity. *Ann. N. Y. Acad. Sci.* **1253**, 16–36 (2012). 22524423 10.1111/j.1749-6632.2012.06517.x PMC3357316

[40] Park, D. D. et al. Metastasis of cholangiocarcinoma is promoted by extended high-mannose glycans. *Proc. Natl Acad. Sci. USA* **117**, 7633–7644 (2020). 32213588 10.1073/pnas.1916498117 PMC7148571

[41] Yadav, V., Panganiban, A. T., Honer Zu Bentrup, K. & Voss, T. G. Influenza infection modulates vesicular trafficking and induces Golgi complex disruption. *Virusdisease* **27**, 357–368 (2016). 28004015 10.1007/s13337-016-0347-3 PMC5142599

[42] Bajaj, R., Warner, A. N., Fradette, J. F. & Gibbons, D. L. Dance of the golgi: understanding golgi dynamics in cancer metastasis. *Cells* **11**, (2022). 10.3390/cells11091484 PMC9102947 35563790

[43] Joshi, G., Bekier, M. E. & Wang, Y. Golgi fragmentation in Alzheimer’s disease. *Front. Neurosci.* **9**, 340 (2015). 26441511 10.3389/fnins.2015.00340 PMC4585163

[44] Dworkin, L. A., Clausen, H. & Joshi, H. J. Applying transcriptomics to studyglycosylation at the cell type level. *iScience* **25**, 104419 (2022). 35663018 10.1016/j.isci.2022.104419 PMC9156939

[45] Sanson, K. R. et al. Optimized libraries for CRISPR-Cas9 genetic screens with multiple modalities. *Nat. Commun.* **9**, 5416 (2018). 30575746 10.1038/s41467-018-07901-8 PMC6303322

[46] Doench, J. G. et al. Optimized sgRNA design to maximize activity and minimize off-target effects of CRISPR-Cas9. *Nat. Biotechnol.* **34**, 184–191 (2016). 26780180 10.1038/nbt.3437 PMC4744125

[47] Watanabe, Y., Aoki-Kinoshita, K. F., Ishihama, Y. & Okuda, S. GlycoPOST realizes FAIR principles for glycomics mass spectrometry data. *Nucleic Acids Res* **49**, D1523–D1528 (2021). 33174597 10.1093/nar/gkaa1012 PMC7778884

