# Supplementary material for: CRISPR screens and lectin microarrays identify high mannose N-glycan regulators
Source: Nat Commun. 2024 Nov 18;15:9970. doi: 10.1038/s41467-024-53225-1 (PMC11574202; doi:10.1038/s41467-024-53225-1)
Supplement: Supplementary file 1 — Supplementary Information [file 41467_2024_53225_MOESM1_ESM.pdf]

Supplementary Fig. 1

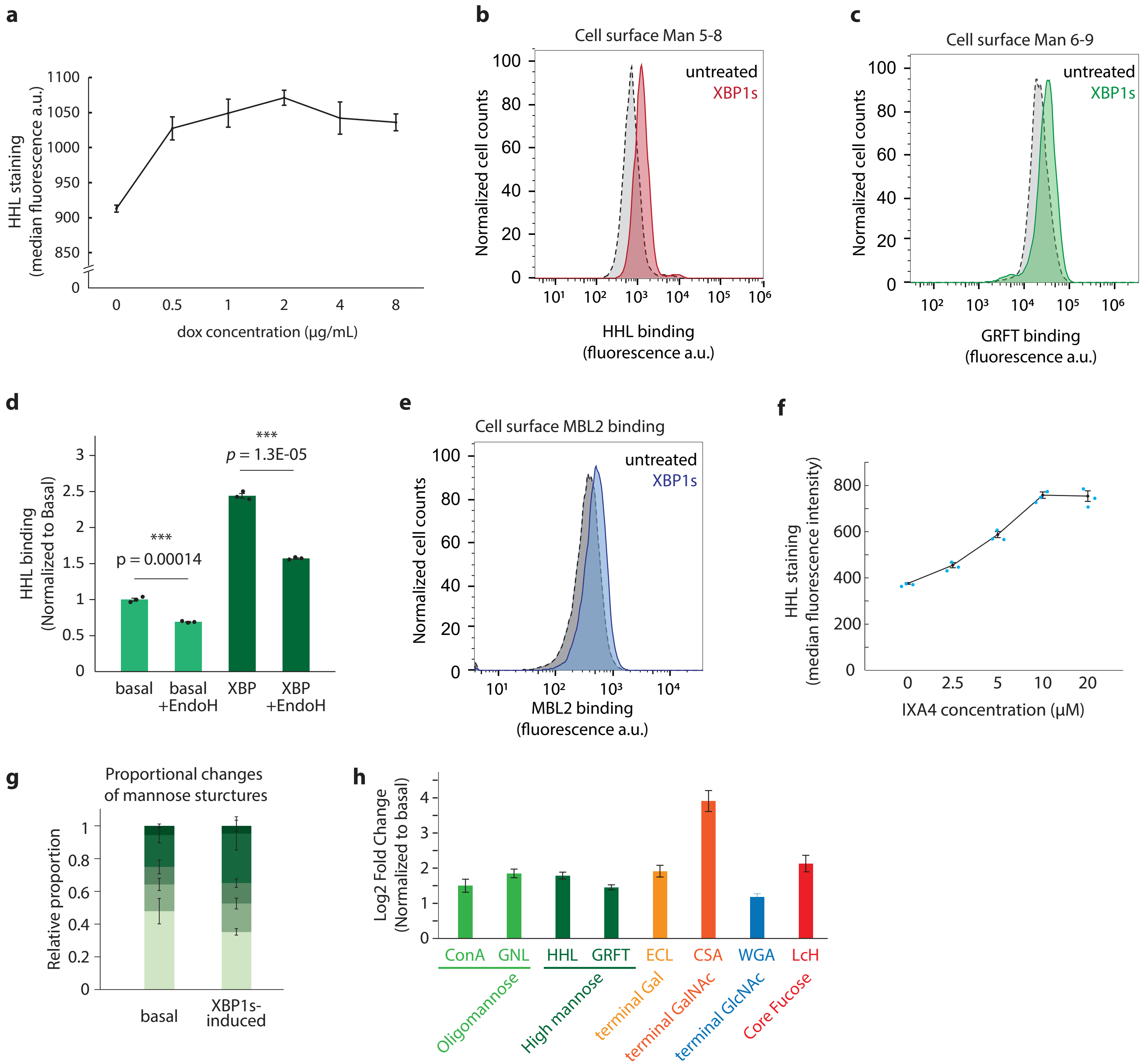

a, Dox-titration of XBP1s-induction. Cell surface HHL-binding is measured by flow cytometry. Data are presented as mean ± s.e.m. and are representative of two independent experiments performed in triplicate with consistent results. **b,** Representative flow cytometry results of lectin binding for HHL as related to Fig. 1c. **c,** Representative flow cytometry results of lectin binding for GRFT as related to Fig. 1C. **d,** HHL staining on cells treated with Endoglycosidase H for 1 hour at 37°C. Data are presented as mean ± s.e.m. and are representative of two independent experiments performed in triplicate with consistent results. **e,** Representative flow cytometry results of lectin binding for MBL2 as related to Fig. 1C. **f,** Cell surface HHL-binding on cells treated with increasing concentration of IXA4. Flow cytometry results are presented as mean ± s.e.m. and are representative of two independent experiments performed in triplicate with consistent results. **g,** Proportional changes of high mannose structures in A549 cells with or without XBP1s-induction, as related to Fig. 1d. The experiment was performed in triplicate, and data is presented as mean ± s.e.m. **h,** Cell surface lectin binding of XBP1s-induced cells normalized to basal control cells. Data are representative of two independent experiments performed in triplicate.

Supplementary Fig. 2

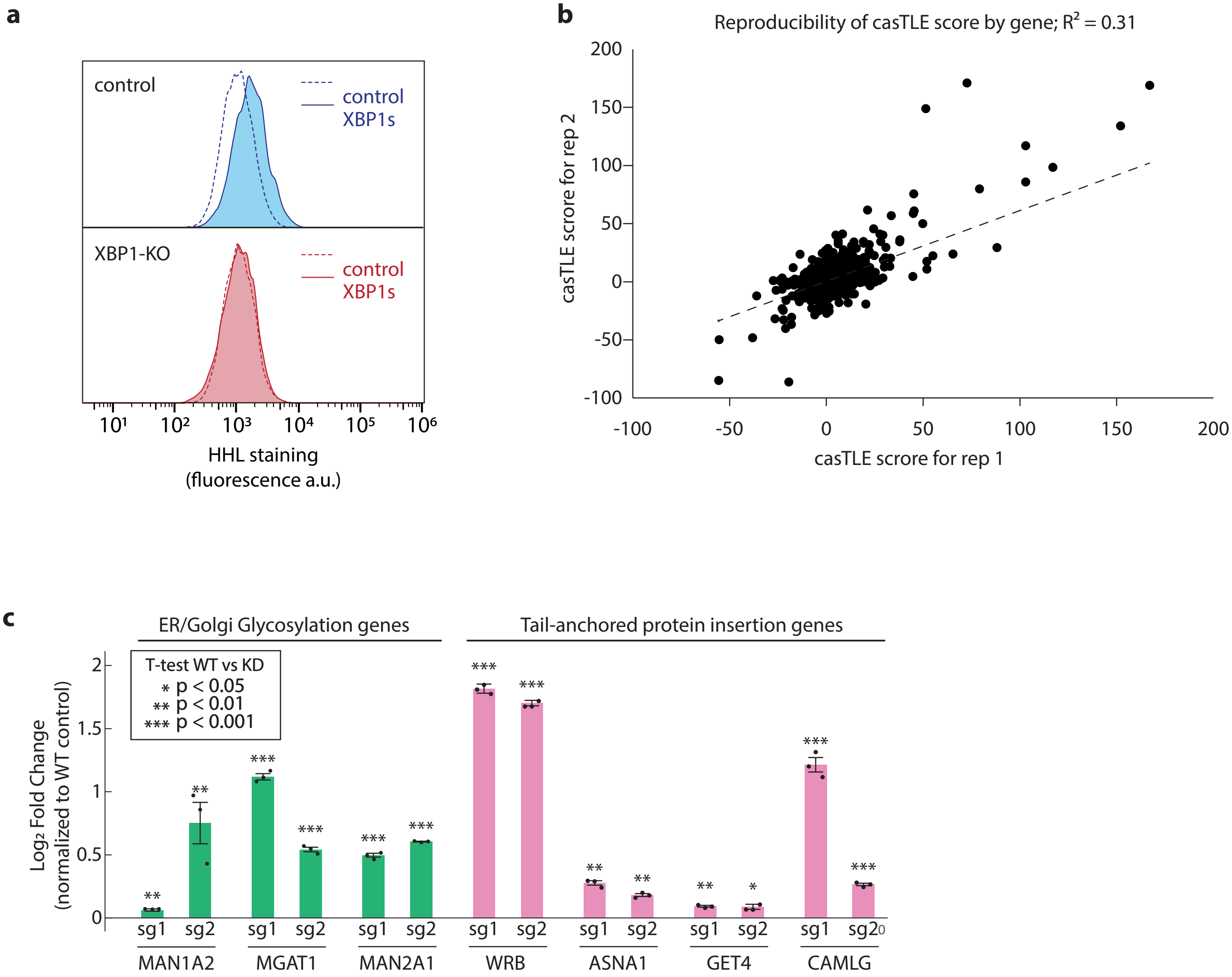

a, Flow cytometry result of HHL binding in A549 wild type control and cells expressing XBP1-targeting sgRNA (XBP1-KO). Data are representative of two independent experiments performed in triplicate. **b,** Reproducibility plot of casTLE scores for the CRISPR-KO genome-wide screen. **c,** Validation of hits in basal A549s using competitive HHL binding assays: Data are presented as mean ± s.e.m. and are representative of two independent experiments performed in triplicate with consistent results.

Supplementary Fig. 3

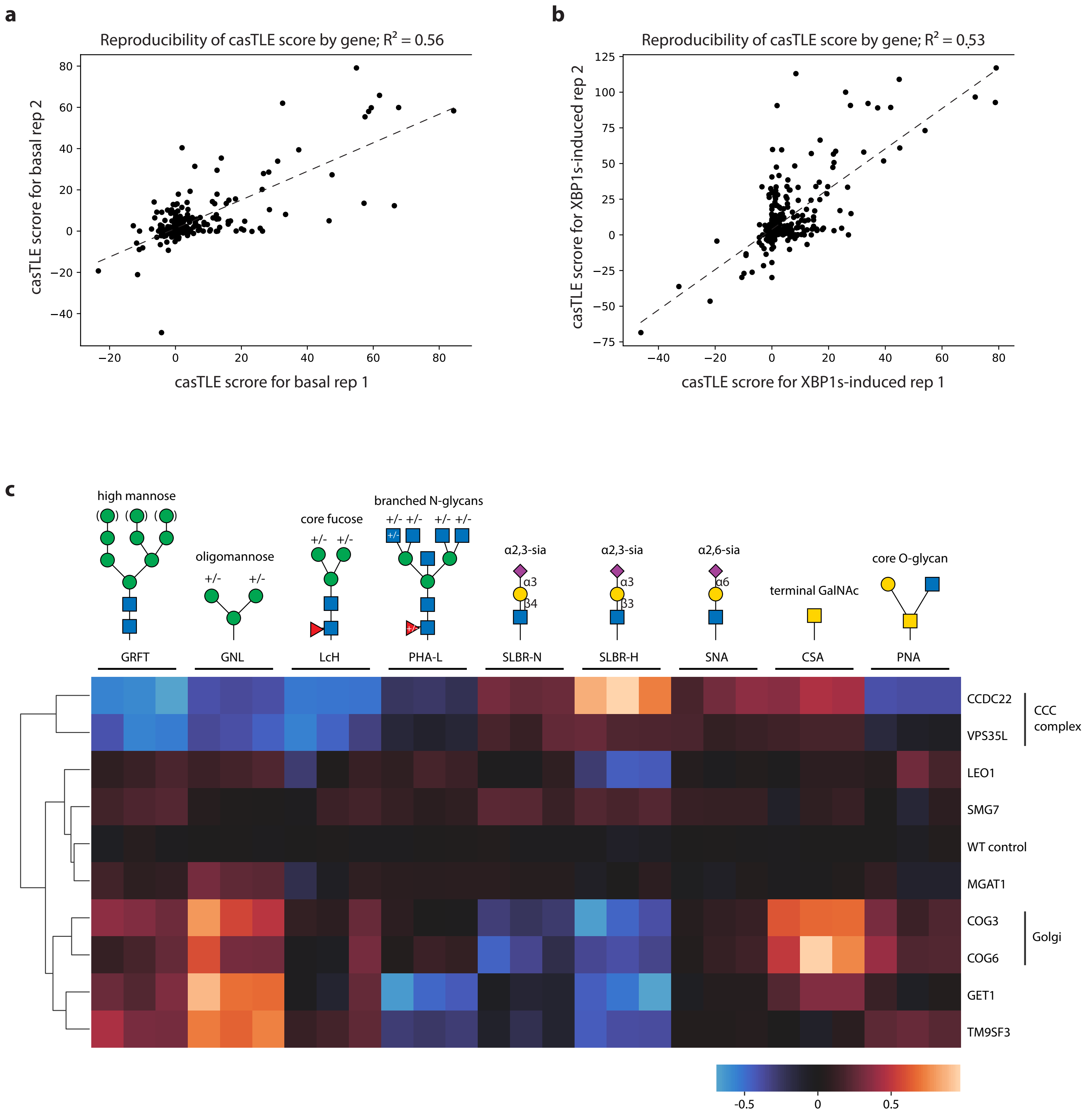

a, Reproducibility plot of casTLE scores for the CRISPRi targeted screen under basal condition. **b,** Reproducibility plot of casTLE scores for the CRISPRi targeted screen under XBP1s-indcued condition. **c,** Clustered heat map representing the results of competitive cell surface lectin binding assay for KD of top screen hits under basal conditions. Lectin binding specificities are indicated. Each column is a replicate and each row represents a different gene knock down.

Supplementary Fig. 4

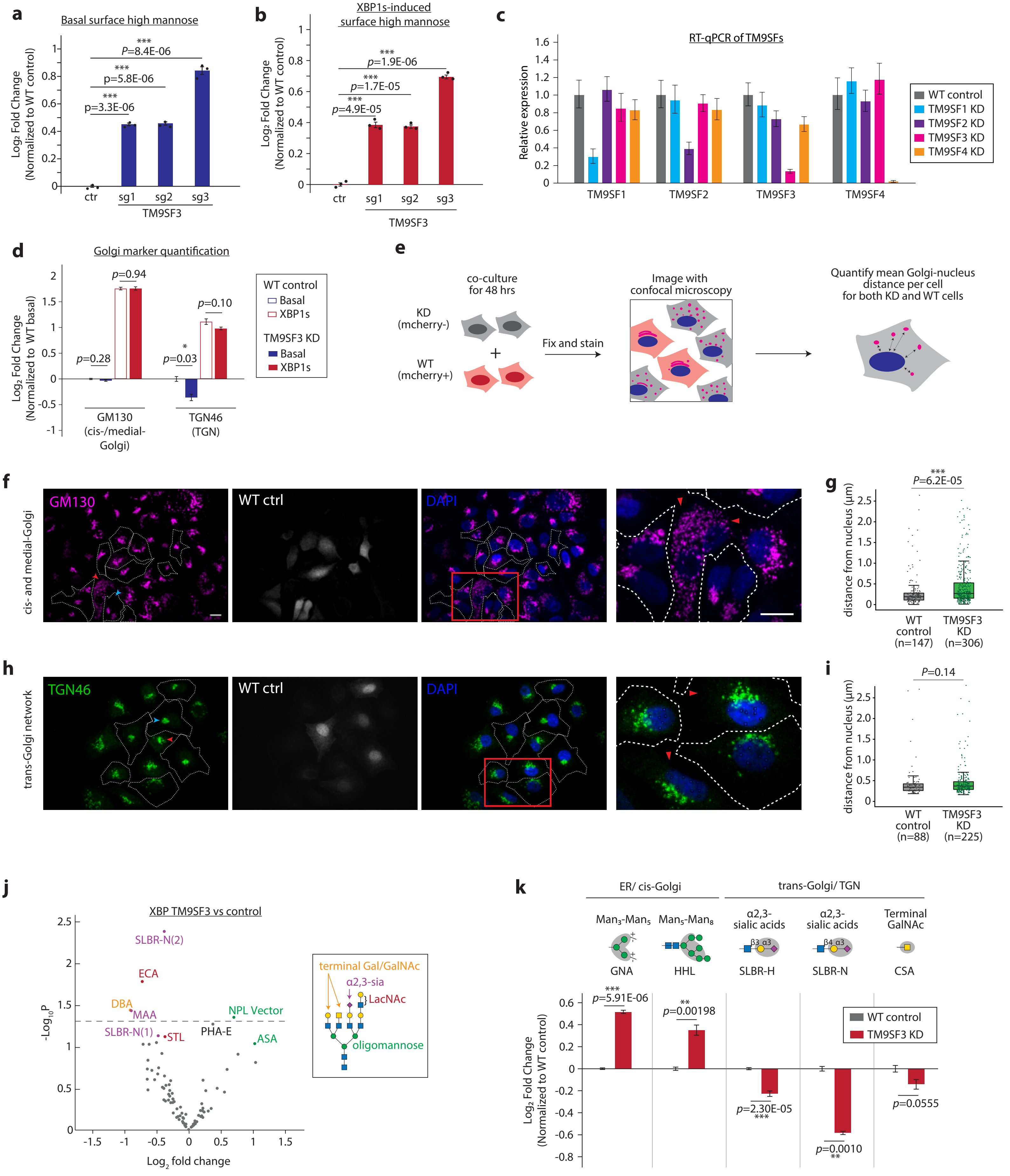

a, Competitive HHL binding assay in A549s with expressing three independent sgRNAs targeting TM9SF3. Data are presented as mean ± s.e.m. and are representative of two independent experiments performed in triplicate. **b,** Competitive HHL binding assay in A549s under basal conditions expressing three independent sgRNAs targeting TM9SF3. Data are presented as mean ± s.e.m. and are representative of two independent experiments performed in triplicate with consistent results. **c,** RT-qPCR for TM9SF family members. Gene expression is normalized to housekeeping genes GAPDH and HRPT1. **d,** Flow cytometry quantification of intracellular staining of GM130 and TGN46 in TM9SF3 knockdown cells under basal and XBP1s-induced conditions compared to wildtype control. Data are presented as mean ± s.e.m. and are representative of three independent experiments performed in triplicate with consistent results. **e,** Schematic for microscopy analysis. Wildtype control cells (mcherry-positive) were cocultured with TM9SF3 knockdown cells (mcherry-negtaive) as in-well internal controls. **f and h,** Representative confocal microscopy images of TM9SF3 knockdown and wildtype control cells, stained with cis-/medial-Golgi marker GM130 (f) or TGN marker TGN46 (h). Wildtype cells are outlined in dotted white lines. Magnified view of the red boxed areas are shown in the right-most column, with red arrows denoting KD cells. Scale bars, 10 μm. Images are representative of two independent experiments performed in triplicate. **g and i,** Quantification of average cis-/medial-Golgi or TGN distances from the nucleus in Extended Data Fig. 4f and 4h, respectively. Each data point represents the mean distance of a single cell. P-value is calculated by Mann-Whiteney U test. **j,** Volcano plot for lectin microarray results of XBP1s-induced A549 cells with TM9SF3 knocked down compared to wild type control. Lectins are color-coded by their glycan-binding specificities. **k,** Competitive cell surface lectin binding assay for TM9SF3 knocked down A549s compared to wildtype control under XBP1s-induced conditions. Lectin binding specificities and the location of where the modification predominately occurs are indicated. Data are presented as mean ± s.e.m. and are representative of two independent experiments performed in triplicate with consistent results.

Supplementary Fig. 5

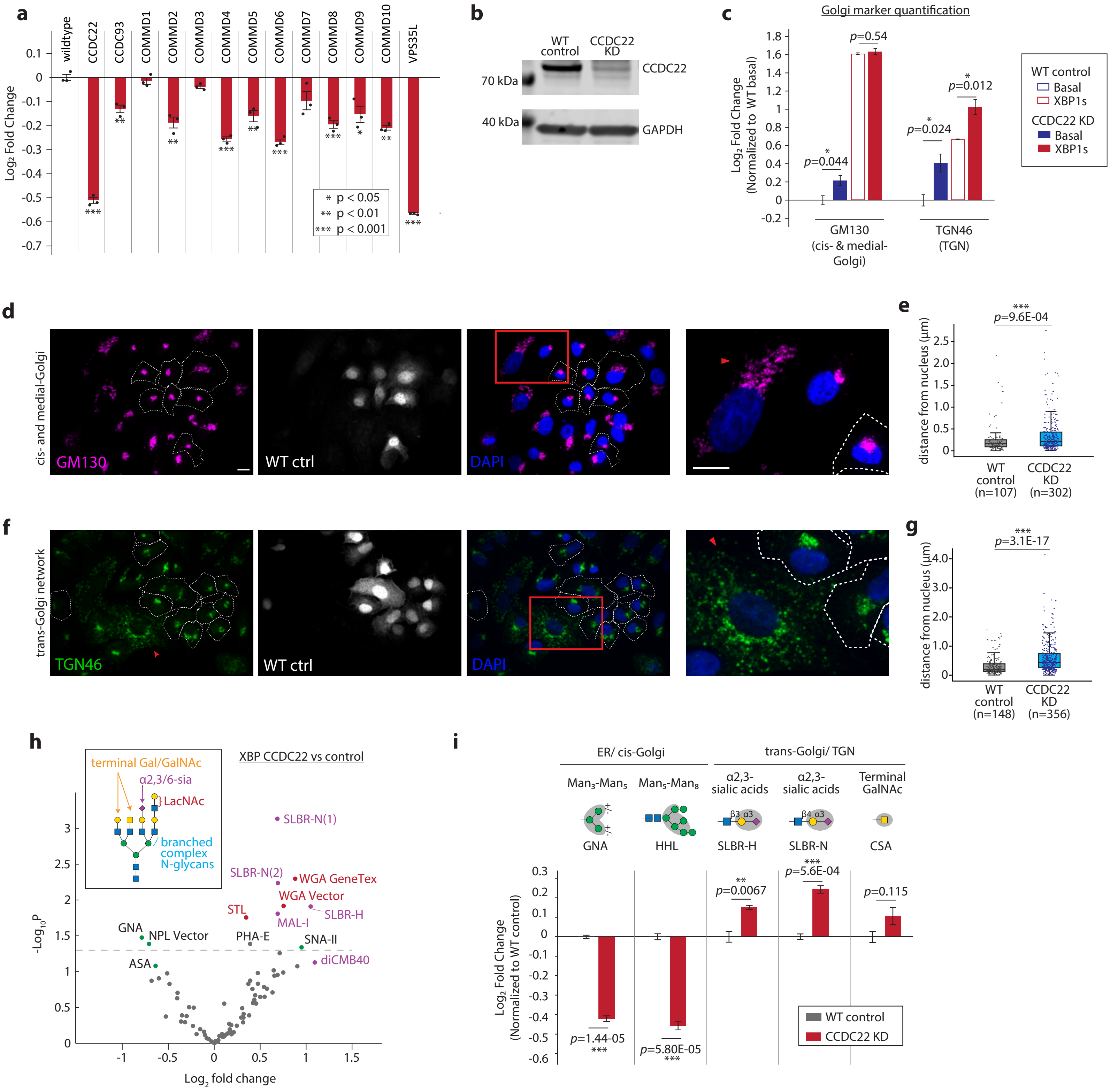

a, Competitive HHL binding assays on A549s under XBP1s-induced conditions for all knock down of all members of the CCC complex. Each gene is knocked down by co-expression of two independent sgRNAs. Data are presented as mean ± s.e.m. and are representative of three independent experiments performed in triplicate with consistent results. **b,** Western blot of CCDC22 knockdown cell line showing expected reduction in CCDC22 protein levels. **c,** Flow cytometry quantification of intracellular staining of GM130 and TGN46 in CCDC22 knockdown cells under basal and XBP1s-induced conditions compared to wildtype control. Data are presented as mean ± s.e.m. and are representative of three independent experiments performed in triplicate with consistent results. **d and f,** Representative confocal microscopy images of CCDC22 knockdown and wildtype control cells, stained with cis-/medial-Golgi marker GM130 (d) or TGN marker TGN46 (f). Wildtype cells are outlined in dotted white lines. Magnified view of the red boxed areas are shown in the right-most column, with red arrows denoting KD cells. Scale bars, 10 µm. Images are representative of two independent experiments performed in triplicate. **e and g,** Quantification of average cis-/medial-Golgi or TGN distances from the nucleus in Extended Data Fig. 5d and 5f, respectively. Each data point represents the mean distance of a single cell. P-value is calculated by Mann-Whiteney U test. **h,** Volcano plot for lectin microarray results of XBP1s-induced A549 cells with CCDC22 knocked down compared to wild type control. Lectins are color-coded by their glycan-binding specificities. **i,** Competitive cell surface lectin binding assay for CCDC22 knocked down A549s compared to wildtype control under XBP1s-induced conditions. Lectin binding specificities and the location of where the modification predominately occurs are indicated. Data are presented as mean ± s.e.m. and are representative of two independent experiments performed in triplicate with consistent results.

Supplementary Fig. 6

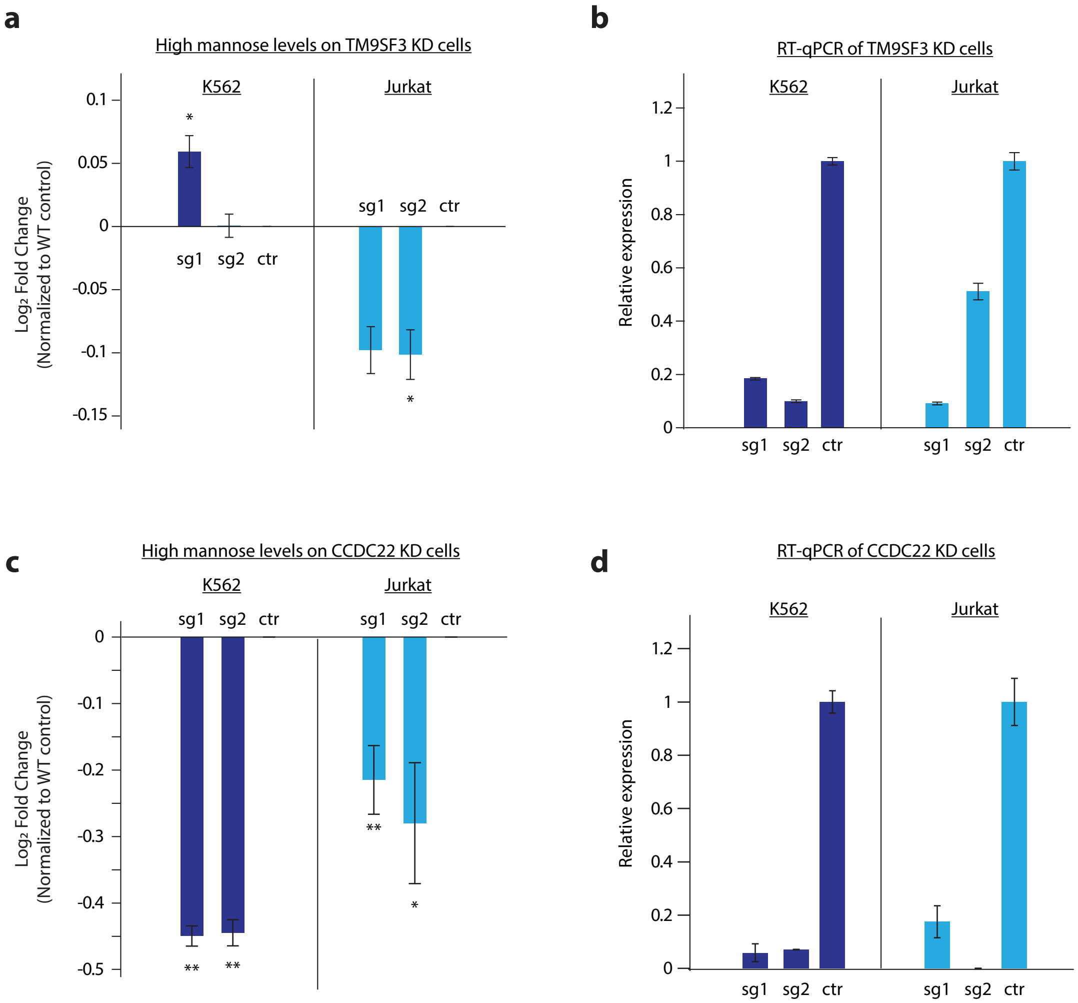

a, Competitive HHL binding assay in K562 and Jurkat cells expressing two independent sgRNAs targeting TM9SF3. Data are presented as mean ± s.e.m. and are representative of three independent experiments performed in triplicate. **b,** RT-qPCR for TM9SF3 in K562 and Jurkat cells. Gene expression is normalized to housekeeping genes GAPDH and HRPT1. **c,** Competitive HHL binding assay in K562 and Jurkat cells expressing two independent sgRNAs targeting CCDC22. Data are presented as mean ± s.e.m. and are representative of three independent experiments performed in triplicate. **d,** RT-qPCR for CCDC22 in K562 and Jurkat cells. Gene expression is normalized to housekeeping genes GAPDH and HRPT1.

Supplementary Fig. 7

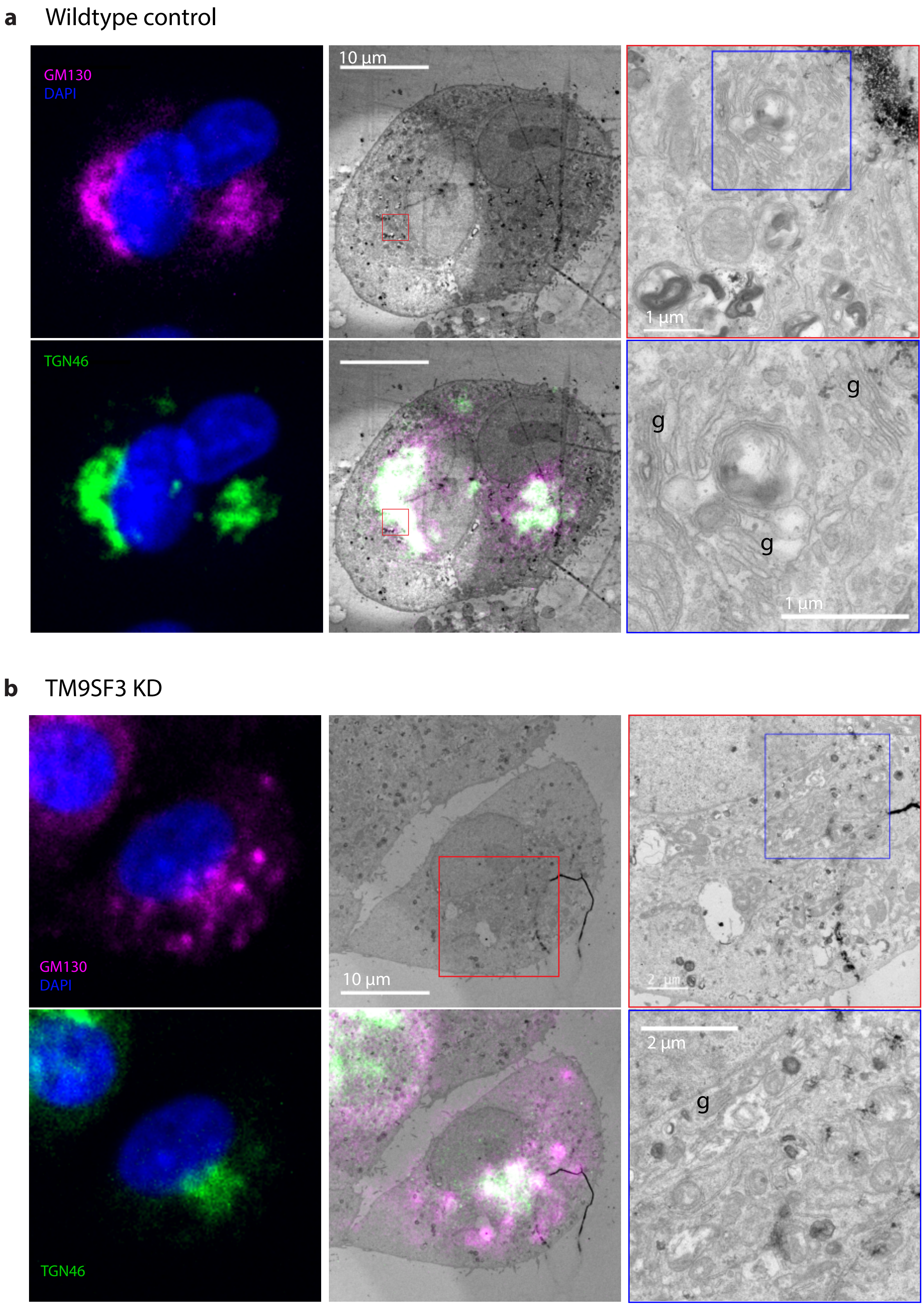

Representative correlative light and electron microscopy microscopy (CLEM) images of **a,** wildtype control and **b,** TM9SF3 knockdown A549 cells. Cells were co-stained with cis-/medial-Golgi marker GM130 and TGN marker TGN46. Magnified views of the red and blue boxed areas are shown in the right-most column. g=Golgi.

Supplementary Fig. 8

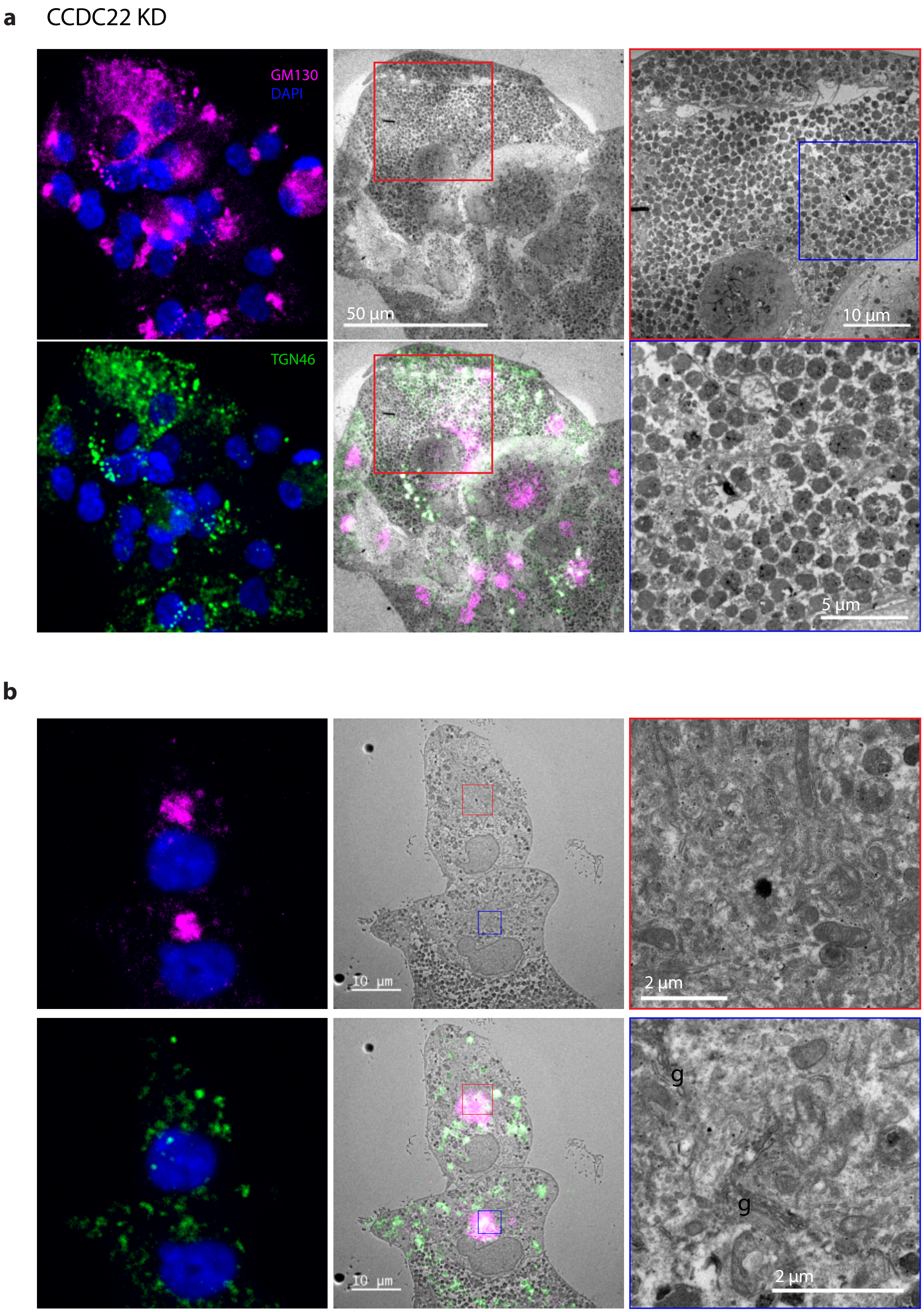

Representative examples of correlative light and eletcron microscopy microscopy (CLEM) images of CCDC22 knockdown A549 cells. **a,** show cells with severe Golgi fragmentation and dispersion phenotype with an accumulation of vesicular structures , while **b,** shows a milder phenotype in which Golgi stacks can still be observed. Cells were co-stained with cis-/medial-Golgi marker GM130 and TGN marker TGN46. Magnified views of the red and blue boxed areas are shown in the right-most column. g=Golgi.

Supplementary Fig. 9

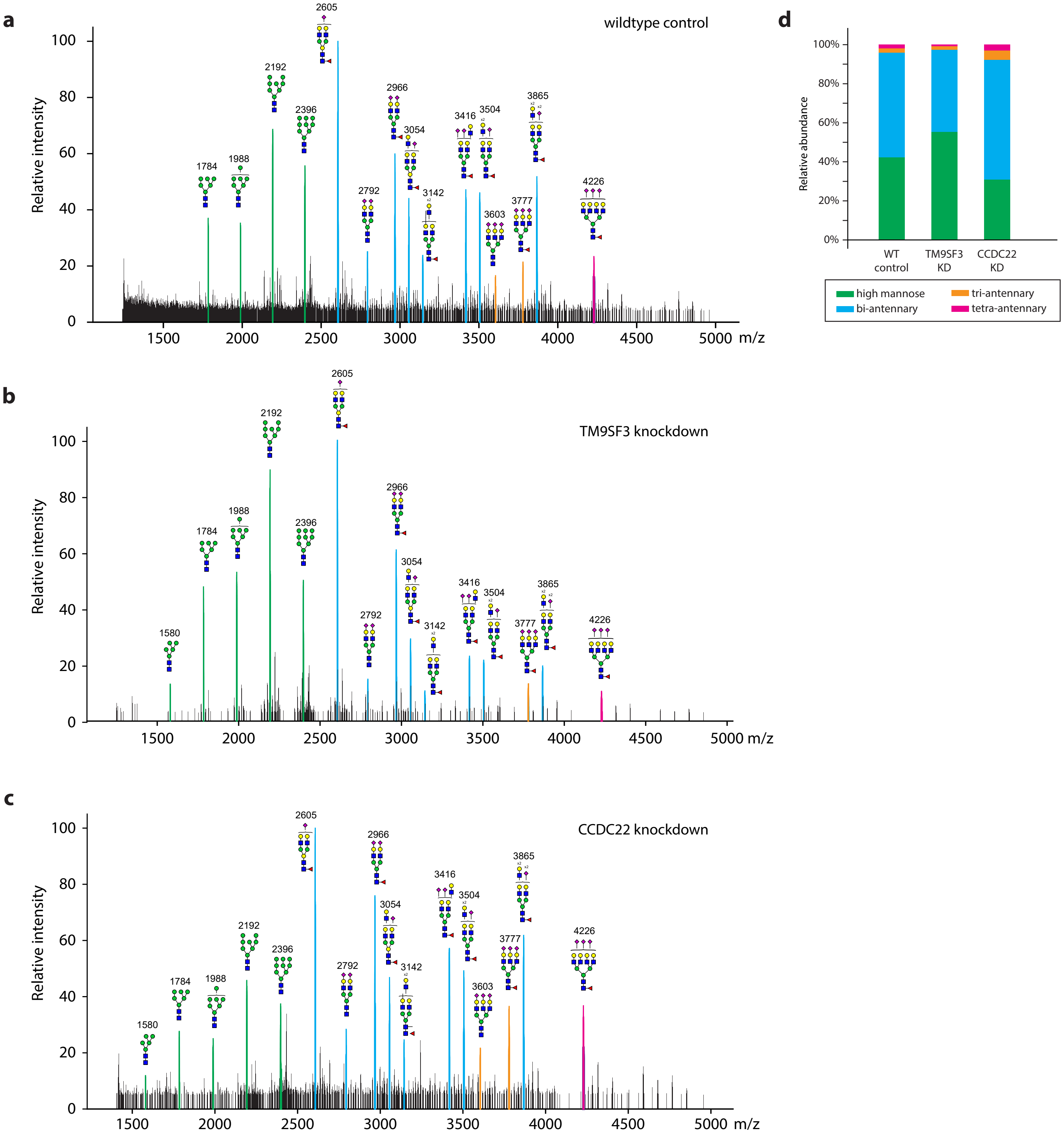

N-glycomics analysis of **a,** wildtype control, **b,** TM9SF3 knockdown, and **c,** CCDC22 knockdown cells. Representative partial MALDI-TOF mass spectra are shown. Only peaks with relative intensity above 3% are shown. Peaks of high mannose, bi, tri, and tetra-antennary strucutres are shown in green, blue, orange, and magenta, repsectively. Structures above a bracket were not unequivocally defined. Putative structures are based on composition, tandem MS, and knowledge of biosynthetic pathways. All molecular ions are [M+Na]+. **d,** Relative abundance of the indicated N-glycan strucutres. Data is presented as mean of two replicates each. Full peak intensity data can be found in Supplementary table 5.

Supplementary Fig. 10

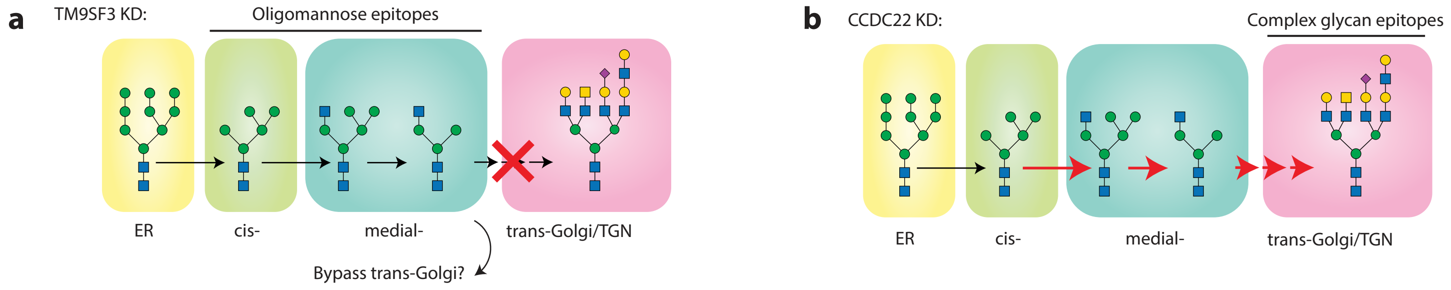

a, Model for how glycosylation is altered in TM9SF3-KD cells: High mannose glycans are converted into oligomannose glycans in the cis- and medial-Golgi despite changes in morphology. However, the final stages of complex glycan synthesis, such as elongation by LacNAc motifs and capping with sialic acids, are inhibited. **b,** Model for how glycosylation is altered in CCDC22-KD cells: Despite the fragmented Golgi morphology, glycans are able to be remodeled from high mannose into complex glycans, perhaps at an enhanced efficiency. Resulting in a more complex N-glycan repertoire at the expense of high mannose glycans.

Supplementary Table 1: Lectin Microarray Information

	Description*
**1. Sample: Glycan-containing sample (e.g. glycan, glycoprotein, cell lysate, cell, glycopeptide, etc.)**	
Description of Sample	Glycoproteins extracted from A549 cells.
Sample preparation protocol	A549 cell pellets are washed with PBS supplemented with protease inhibitor cocktail. The solutions are sonicated on ice until homogenous and then ultracentrifuged to collect pellet prior to labeling.
Labeling protocol for sample detection	Samples are labelled with Alexa Fluor 555-NHS (Thermo Fisher).
Two-color reference (if used)	A pooled reference of all samples labeled with Alexa Fluor 647-NHS (Thermo Fisher).
Assay protocol	Lectin microarrays are blocked with blocking buffer (50 mM ethanolamine and 100 mM boric acid) for one hour at room temperature. Slides are rinsed once with PBST (0.01%) and once with PBS for 5 minutes each, then dried using a slide spinner. Each slide is mounted on a 24-well format hybridization cassette (Arrayit), in which each well contains a subarray. To each well, 5 µg of sample and pooled reference are added, then diluted with PBST to reach the final volume (100uL) and concentration (0.005%). Slides are incubated on an orbital shaker for one hour at room temperature in the dark. After hybridization, arrays are washed with PBST (0.01%) once for one minute, then once for five minutes. Arrays are lastly washed once with PBS for 1 minute, then once for ten minutes. Once finished, slides are removed from the cassette and briefly immersed in ultrapure water, then dried using a slide spinner.
**2. Lectin Library**	
General description of the lectin library used in the array	Lectin microarrays are generated in house.
List of lectins and/or glycan-binding proteins, their source, concentration, and buffer	Please see **Supplemental Table S###**.
Modification of lectins	N/A
**3. Immobilization Surface; e.g., Microarray Slide**	

Immobilization surface	Nexterion Slide H Barcoded 3D Hydrogel Coated.
Manufacturer	Schott North America.
Custom preparation of the surface	N/A
**4. Array Production**	
Description of Arrayer	Nano-Plotter 2.1 piezoelectric printer (GeSim, Germany) with cooled microwell plate holder and cooled printing deck.
Lectin deposition	Triplicates of each lectin are printed onto each subarray.
Printing conditions	Dilute lectins to the pre-determined concentrations in the print buffer (final concentration of print buffer: 1 mM monosaccharide in PBS, 5 ng/mL Atto 532; Please see Supplemental Table 1 for the concentrations of lectins). Load the mixed solution to the microplate. Before printing, check the humidity of the print chamber. The humidity should be kept around 50% throughout the entire print. Ensure both microwell plate holder and printing deck are cooled. Adjust the cooling temperature based on ambient temperature and the temperature of the cooled slide deck surface, preventing moisture building up inside the print chamber. Once printing is complete, allow the slides to dry for at least one hour.
Array layout	Each microarray contains 24 subarrays (3 columns and 8 rows). In each subarray, triplicates of a lectin are printed, with six lectins in each row. The number of columns is 18, and the row number depends on how many lectin probes are printed on the arrays (i.e. 132 lectins require 22 rows).
Quality control	The well-characterized glycoproteins glycophorin, ovalbumin, albumin, 1:1 A549:HEK293T cell lysate, human serum, fetuin, and RNase B are used for quality assurance of the printed microarrays.
**5. Detector and Data Processing**	
Instrument (scanner, flow cytometer)	Fluorescent Slide Scanner GenePix 4400A (Molecular Devices).
Instrument settings	A low resolution preview scan of the slide is performed to adjust photomultiplier tube (PMT) gain for each channel (Alexa Fluor 555: 532nm, Alexa Fluor 647: 635nm) so that the signals are not saturated and within the linear detection range. After adjusting PMT gain, slides are scanned in each channel at 5 µm resolution.
Image analysis software	GenePix Pro 7 (Molecular Devices).
Data processing and statistical analysis	Extracted data is processed for quality checks using Grubbs outlier test with $\alpha = 0.05$. Log2 values of the average signals are median-normalized over the individual subarray in each channel.

6. Lectin Microarray Data Presentation	
Data presentation and interpretation	Hierarchical clustering of the processed data is performed using Pearson Correlation coefficient, and volcano plots generated using R (v3.6.1) and RStudio (Build 576).
**7. Data Location**	
Data Location	https://doi.org/10.7303/syn52845352

Lectins used in microarrays

Lectin	Species/Origin	Print Conc. (µg/mL)	Rough Specificity /Inhibitory monosaccharide	Vendor/Source
AAL	*Aleuria aurantia*	2000	Fucose	Medicago/Vector
ACA	*Amaranthus Caudatus*	2000	Gal-β1,3-GalNAc	EY
AIA	*Artocarpus integrifolia*	2000	β1,3-GalNAc	EY/Glycomatrix/Vector
AMA	*Allium moly*	2000	Oligo mannose	Vector
Anti-B.G. Lewis A	MAb mouse IgG1 [7LE]	undiluted	Lewis A	Abcam/Thermo Fisher
Anti-B.G. Lewis B	MAb mouse IgM [2-25LE]	undiluted	Lewis B	Abcam/Sigma
Anti-B.G. Lewis Y	MAb mouse IgM [F3]	undiluted	Lewis Y	Abcam
Anti-Lewis X [P12]	MAb mouse IgM [P12]	undiluted	Lewis X	Sigma
Anti-Sialyl Lewis A	MAb mouse IgG1 [GT933]	undiluted	Sialyl Lewis A	GeneTex
Anti-Sialyl Lewis X	MAb mouse IgM [258-12767]	undiluted	Sialyl Lewis X	GeneTex
AOL	*Aspergillus oryzae*	2000	Fucose	TCI America
ASA	*Allium sativum*	2000	Mannose	EY
BambL	*Burkholderia ambifaria*	2000	Fucose	Generated in house
BanLec H84T	*Musa acuminate*	2000	High Mannose	Gift from Dr. David Markovitz
BC2L-A	*Burkholderia cenocepacia*	2000	Mannose	Elicityl
BPA/BPL	*Bauhinia purpurea*	2000	β-Gal / β-GalNAc	Vector
CA	*Colchicum autumnale*	2000	Bi-antennary N-linked glycans	EY
ConA	*Canavalia ensiformis*	2000	Tri-mannose core	Thermo Fisher/Vector

DBA	*Dolichos biflorus*	2000	GalNAc	Vector
diCBM40	engineered NanI from *Clostridium perfringens*	1500	α Sialylation	Generated in house
DSA	*Datura stramonium*	2000	LacNAc	Vector
ECA	*Erythrina cristagalli*	2000	LacNAc	EY/Vector
GNA/GNL	*Galanthus nivalis*	2000	Oligo mannose	Sigma/Vector
GS/GSL-I	*Griffonia simplicifoia-I*	2000	α-Gal / Lac	Vector
GS/GSL-II	*Griffonia simplicifoia-II*	2000	GlcNAc	Vector
H84T	*Banana lectin*	1000	High mannose	Gift from Dr. David Markovitz
HHL	*Hippeastrum hybrid*	2000	Oligo/High mannose	BioWorld
HPA	*Helix pomatia*	2000	Blood Group A	Sigma
LcH	*Lens culinaris*	2000	Core Fucose	Aniara Diagnostica/EY/Medicago
LEA/LEL	*Lycopersicon esculentum*	2000	GlcNAc	Vector
LTL Lotus	*Lotus tetragonolobus*	2000	Fucose	EY/Vector
MAA/MAL-I	*Maackia amurensis-I*	2000	Sialylation/Sulfation	EY/Vector
MAA/MAL-II	*Maackia amurensis-II*	2000	Sialylation/Sulfation	Vector
MNA-G	*Morus nigra Morniga G*	2000	GalNAc	EY
MNA-M	*Morus nigra Morniga M*	2000	Oligo mannose / Gal	EY
MPA/MPL	*Maclura pomifera*	2000	β1,3-GalNAc	Vector
NPA/NPL	*Narcissus pseudonarcissus*	2000	Oligo mannose	EY/Vector
PA-IIL	*Pseudomonas aeruginosa*	2000	Fucose	Elicityl
PHA-E	*Phaseolus vulgaris Erythroagglutinin*	2000	Bisecting GlcNAc	Sigma/Vector
PHA-L	*Phaseolus vulgaris Leukoagglutinin*	2000	β1,6 Branching N-Link glycans	Medicago/Vector
PNA	*Arachis hyogaea*	2000	Gal-β1,3-GalNAc	EY/Vector
Recombinant Protein A	*Staphylococcus aureus*	2000	Immunoglobulins	Thermo Fisher
Recombinant Protein G	*Streptococcus* Group G	2000	Immunoglobulins	Thermo Fisher
Recombinant Protein L	*Peptostreptococcus magnus*	2000	Immunoglobulins	Thermo Fisher

PSA	*Pisum sativum*	2000	Core Fucose	Glycomatrix /Vector
PSL	*Polyporus squamosus*	2000	α2,6 sialylation	EY
PTA/PTL-II	*Psophocarpus tetragonolobus-II*	2000	α2 Fucose	Glycomatrix
RCA120	*Ricinus Communis Agglutinin I*	2000	Gal / Lac	Vector
rGRFT	*recombinant Griffithsin*	1700	High mannose	Gift from Dr. Barry O'Keefe
Ricin B Chain	*Ricinus communis*	2000	Gal	Vector
RPA	*Robinia pseudoacacia*	2000	Multiantennary N-glycans with bisecting GlcNAc	EY
SBA	*Glycine max*	2000	LacdiNAc	Vector
SLBR-B	*Streptococcus gordonii M99*	2300	α2,6 sialylation	Generated in house
SLBR-H	*Streptococcus gordonii DL1*	2000	α2,3 sialylation	Generated in house
SLBR-N	*Streptococcus gordonii UB10712*	2000	α2,3 sialylation	Generated in house
SNA	*Sambucus nigra*	2000	α2,6 sialylation	EY/Vector
SNA-II	*Sambucus nigra-II*	2000	α2 Fucose /oligo mannose	EY
STL	*Solanum tuberosum Agglutinin*	2000	LacNAc	Vector
TJA-II	*Trichosanthes japonica-II*	2000	α2 Fucose	Aniara Diagnostica/Medicago
TL	*Tulipa sp.*	2000	GlcNAc	EY
UDA	*Urtica dioica*	2000	GlcNAc / Oligo mannose	EY
UEA-I	*Ulex europaaeus-I*	2000	α2 Fucose	BioWorld/GeneTex/Vector
UEA-II	*Ulex europaaeus-II*	2000	GlcNAc	EY/Vector
VVA	*Vicia villosa*	2000	Terminal GalNAc	EY/Vector
WFA	*Wisteria floribunda*	2000	GalNAc-β1,4	Vector
WGA	*Triticum vulgare*	2000	GlcNAc	GeneTex/Vector
