# Supplementary material for: CRISPR screens and lectin microarrays identify high mannose N-glycan regulators
Source: Nat Commun. 2024 Nov 18;15:9970. doi: 10.1038/s41467-024-53225-1 (PMC11574202; doi:10.1038/s41467-024-53225-1)
Supplement: Supplementary file 2 — Description of Additional Supplementary Information [file 41467_2024_53225_MOESM2_ESM.docx]

**Description of Additional Supplementary Files**

File Name: Supplementary Data 1

Description: Lectin microarray results for basal vs XBP1s-induced cells

File Name: Supplementary Data 2

Description: FACS Genome-wide screening results

File Name: Supplementary Data 3

Description: MACS targeted screening results

File Name: Supplementary Data 4

Description: Lectin microarray results for wildtype, TM9SF3-KD, and CCDC22-KD cells

File Name: Supplementary Data 5

Description: N-glycomics peak-intensities for wildtype, TM9SF3-KD, and CCDC22-KD cells

File Name: Supplementary Data 6

Description: sgRNA sequences for targeted library

File Name: Supplementary Data 7

Description: Primer designs for library sequencing

File Name: Supplementary Data 8

Description: sgRNA sequences for validations

File Name: Supplementary Data 9
Description: qPCR sequences
