# Supplementary material for: CRISPR screens and lectin microarrays identify high mannose N-glycan regulators
Source: Nat Commun. 2024 Nov 18;15:9970. doi: 10.1038/s41467-024-53225-1 (PMC11574202; doi:10.1038/s41467-024-53225-1)
Supplement: Supplementary file 12 — Reporting Summary [file 41467_2024_53225_MOESM12_ESM.pdf]

nature portfolio

Corresponding author(s): C. Kimberly Tsui, Andrew Dillin

Last updated by author(s): Sep 26, 2024

Reporting Summary

Nature Portfolio wishes to improve the reproducibility of the work that we publish. This form provides structure for consistency and transparency in reporting. For further information on Nature Portfolio policies, see our Editorial Policies and the Editorial Policy Checklist.

Statistics

For all statistical analyses, confirm that the following items are present in the figure legend, table legend, main text, or Methods section.

n/a	Confirmed	
☐	☒	The exact sample size (*n*) for each experimental group/condition, given as a discrete number and unit of measurement
☐	☒	A statement on whether measurements were taken from distinct samples or whether the same sample was measured repeatedly
☐	☒	The statistical test(s) used AND whether they are one- or two-sided<br>*Only common tests should be described solely by name; describe more complex techniques in the Methods section.*
☒	☐	A description of all covariates tested
☐	☒	A description of any assumptions or corrections, such as tests of normality and adjustment for multiple comparisons
☐	☒	A full description of the statistical parameters including central tendency (e.g. means) or other basic estimates (e.g. regression coefficient) AND variation (e.g. standard deviation) or associated estimates of uncertainty (e.g. confidence intervals)
☐	☒	For null hypothesis testing, the test statistic (e.g. *F*, *t*, *r*) with confidence intervals, effect sizes, degrees of freedom and *P* value noted<br>*Give P values as exact values whenever suitable.*
☒	☐	For Bayesian analysis, information on the choice of priors and Markov chain Monte Carlo settings
☒	☐	For hierarchical and complex designs, identification of the appropriate level for tests and full reporting of outcomes
☐	☒	Estimates of effect sizes (e.g. Cohen's *d*, Pearson's *r*), indicating how they were calculated

Our web collection on statistics for biologists contains articles on many of the points above.

Software and code

Policy information about availability of computer code

Data collection	ThermoFisher Attune NxT, BD Fortessa and Aria were used flow cytometry and FACS. iQ3 acquisition software (Andor) was used for confocal microscopy imaging. Molecular Devices Genepix 4400A was used for lectin microarray data acquisition. Odyssey CLx imaging system (LI-COR) was used for Western blot imaging.
Data analysis	For screen analysis we used custom Python scripts available at https://bitbucket.org/dmorgens/castle (see methods section, 'FACS-based CRISPR-deletion screen for high mannose regulators'). Flowjo 9.9 was used for flow cytometry analyses. Genepix Pro 7 was used for lectin microarray analysis. Image J and Cell Profiler was used for confocal image analysis.

For manuscripts utilizing custom algorithms or software that are central to the research but not yet described in published literature, software must be made available to editors and reviewers. We strongly encourage code deposition in a community repository (e.g. GitHub). See the Nature Portfolio guidelines for submitting code & software for further information.

Data

Policy information about availability of data

All manuscripts must include a data availability statement. This statement should provide the following information, where applicable:

- Accession codes, unique identifiers, or web links for publicly available datasets
- A description of any restrictions on data availability
- For clinical datasets or third party data, please ensure that the statement adheres to our policy

The complete sgRNA counts are deposited in FigShare and the complete results of all screens are in Supplementary Data 2 and 3. All sgRNA sequences used in

targeted screens are in Supplementary data 6, and an sgRNAs for independent validation experiments are in Supplementary Tables 8. Complete lectin microarray data are in Supplementary Data 4.

Research involving human participants, their data, or biological material

Policy information about studies with human participants or human data. See also policy information about sex, gender (identity/presentation), and sexual orientation and race, ethnicity and racism.

Reporting on sex and gender	NA
Reporting on race, ethnicity, or other socially relevant groupings	NA
Population characteristics	NA
Recruitment	NA
Ethics oversight	NA

Note that full information on the approval of the study protocol must also be provided in the manuscript.

Field-specific reporting

Please select the one below that is the best fit for your research. If you are not sure, read the appropriate sections before making your selection.

[x] Life sciences [ ] Behavioural & social sciences [ ] Ecological, evolutionary & environmental sciences

For a reference copy of the document with all sections, see nature.com/documents/nr-reporting-summary-flat.pdf

Life sciences study design

All studies must disclose on these points even when the disclosure is negative.

Sample size	For the screens, two independent replicate screens were performed, which are sufficient for screening technologies as previously reported. For lectin microarrays, 3-4 independent replicates were performed, which are sufficient for lectin microarray studies in cell lines as previously reported.
Data exclusions	No data was excluded.
Replication	Once experiments and procedures were fully optimized, all attempts at replication were successful. Number of replicates for each experiment is indicated in figure legends.
Randomization	Randomization was not performed. Knock out clones were allocated into experimental groups based on their genotype. Negative control cells were grown together with experimental cells as control.
Blinding	Blinding was performed in assessing confocal microscopy images. Other experiments were not blinded.

Reporting for specific materials, systems and methods

We require information from authors about some types of materials, experimental systems and methods used in many studies. Here, indicate whether each material, system or method listed is relevant to your study. If you are not sure if a list item applies to your research, read the appropriate section before selecting a response.

Materials & experimental systems

n/a	Involved in the study	
[ ]	[x]	Antibodies
[ ]	[x]	Eukaryotic cell lines
[x]	[ ]	Palaeontology and archaeology
[x]	[ ]	Animals and other organisms
[x]	[ ]	Clinical data
[x]	[ ]	Dual use research of concern
[x]	[ ]	Plants

Methods

n/a	Involved in the study	
[x]	[ ]	ChIP-seq
[ ]	[x]	Flow cytometry
[x]	[ ]	MRI-based neuroimaging

Antibodies

Antibodies used	mouse anti-GM130 (1:250), BD Biosciences 610822<br>rabbit anti-TGN46 (1:500), Proteintech 13573-1-AP<br>rabbit anti-CCDC22 (1:500), Proteintech 16636-1-AP
Validation	mouse anti-GM130 (1:250), BD Biosciences 610822 validation: P. Marra et al., "The GM130 and GRASP65 Golgi proteins cycle through and define a subdomain of the intermediate compartment", Nat Cell Biol 2001<br><br>rabbit anti-TGN46 (1:500), Proteintech 13573-1-AP validation: X. Wei et al,., "Host RAB11FIP5 protein inhibits the release of Kaposi's sarcoma-associated herpesvirus particles by promoting lysosomal degradation of ORF45", Plos Pathogens 2020<br><br>rabbit anti-CCDC22 (1:500), Proteintech 16636-1-AP validation: KE. McNally et al., "Retriever is a multiprotein complex for retromer-independent endosomal cargo recycling", Nat Cell Biol 2017

Eukaryotic cell lines

Policy information about cell lines and Sex and Gender in Research

Cell line source(s)	Cell lines used:<br>A549 (ATCC CCL-185), obtained from UC Berkeley Cell Culture Facility<br>K562 (ATCC CCL-243), obtained from UC Berkeley Cell Culture Facility<br>Jurkat(ATCC TIB-152), obtained from UC Berkeley Cell Culture Facility
Authentication	UC Berkeley Cell Culture Facility obtained the cell lines directly from ATCC and performed authentication then. No additional authentication was performed.
Mycoplasma contamination	Cell cultures were routinely tested and found negative for mycoplasma infection (MycoAlert, Lonza)
Commonly misidentified lines<br>(See ICLAC register)	The cell lines used in this study is not in the database of commonly misidentified cell lines.

Plants

Seed stocks	NA
Novel plant genotypes	NA
Authentication	NA

Flow Cytometry

Plots

Confirm that:

- [x] The axis labels state the marker and fluorochrome used (e.g. CD4-FITC).
- [x] The axis scales are clearly visible. Include numbers along axes only for bottom left plot of group (a 'group' is an analysis of identical markers).
- [x] All plots are contour plots with outliers or pseudocolor plots.
- [x] A numerical value for number of cells or percentage (with statistics) is provided.

Methodology

Sample preparation	Live or fixed and permeabilized cells were collected and washed in cold dPBS, stained on ice for 60 minutes using fluorescently labeled lectins. Cells were then washed 3x with cold dPBS and analyzed by flow cytometry. (See methods "Flow cytometry analysis of lectins and MBL2 binding" and "Intracellular staining and flow cytometry quantification of GM130 and TGN46".
Instrument	ThermoFisher Attune NxT
Software	ThermoFisher Attune NxT software and FlowJo (v.10)

Cell population abundance	All analyzed samples are pure samples that has undergone identical staining procedure.
Gating strategy	Cells were first gated on FSS/SCC for live cells. Unstained cells and cells incubated with free fluorophore was used to determine the boundaries between negative and positive populations.

☒ Tick this box to confirm that a figure exemplifying the gating strategy is provided in the Supplementary Information.
