# Supplementary material for: CRISPR screens and lectin microarrays identify high mannose N-glycan regulators
Source: Nat Commun. 2024 Nov 18;15:9970. doi: 10.1038/s41467-024-53225-1 (PMC11574202; doi:10.1038/s41467-024-53225-1)
Supplement: Supplementary file 13 — Source Data [file 41467_2024_53225_MOESM13_ESM.pdf]

nature portfolio

Peer Review File

CRISPR screens and lectin microarrays identify high mannose N-glycan regulators

Corresponding Author: Dr Andrew Dillin

This manuscript has been previously reviewed at another journal. This document only contains reviewer comments, rebuttal and decision letters for versions considered at Nature Communications.

Version 0:

Reviewer comments:

Reviewer #2

(Remarks to the Author)
This manuscript focuses on using CRISPR genomic screening to identify genes that regulate presentation of mannose-binding glycans on the cell surface. I previously reviewed this manuscript and thank the authors for their detailed response to my comments. As I said before, the approach is potentially powerful and the authors present some interesting preliminary findings. However, I still feel that the findings of the manuscript are too mechanistically under-developed for publication in its current form. While the authors have assuaged some of my concerns, the majority of my comments have not been sufficiently addressed to change my initial opinion.

Firstly, I still have doubts about the impact and novelty of their findings. To re-state my original comment more precisely, I do not think it is surprising a priori that disrupting a Golgi protein like TM9SF3 would alter cell-surface glycosylation. This has been established in several previous CRISPR screening studies. It would also be intuitively expected given the importance of the Golgi Apparatus in mediating glycosylation in the secretory pathway. My question regarding the biological significance of this finding is largely unaddressed. Is TM9SF3 an essential core structural component of the Golgi machinery that is essential for glycosylation and secretion in all tissues/cell types, like members of the ALG or SEC gene families? Or is it more like a sialyl- or fucosyl-transferase, whose expression/activity is dynamically regulated so as to finely modulate the precise character of protein glycosylation? To me this is the key question to determine how interesting their findings are, and the basic microscopy experiments performed by the authors do not address this point. While they generally comment on the regulation of cell-surface mannose expression in disease, they can’t point to any real link between TM9SF3 and these biological functions. This limitation diminishes my enthusiasm for this study. They also have not attempted to study their hit genes in different cell models, which might help address some of these points.

Secondly, I have questions regarding the exclusive focus on lectins to characterize glycosylation on their cell line. Lectin arrays are an amazing technology and are useful for many applications, in particular when working with precious samples where the amount of cell material available may be too limited for MS analysis. However, there are advantages to MS-based glycomics in terms of reproducibility and structural resolution. Such techniques can very feasibly be applied to proliferating cell lines and would be extremely helpful in interpreting and validating some of the authors findings. For example, knockout of many of their hits seem to reduce both complex (L-PHA) and high mannose N-linked glycosylation, which is somewhat counter-intuitive to me. A global MS profiling dataset of at least some gene knockouts would help clear up exactly what the impact of gene knockout is on glycosylation. Mass Spec glycomics analysis is available as a service through several NIH-funded core facilities, and I don’t think it’s unreasonable to expect some of this characterization when working with cultured cells.

Without addressing these two major points, I’m afraid my opinion on this manuscript remains unchanged from original submission.

Reviewer #3

(Remarks to the Author)
The authors have adequately addressed most of the questions. I will look forward to seeing the manuscript in print.

Reviewer #4

(Remarks to the Author)
Regarding the paper submitted to Nat Comm by Tsui et al, at the request of the editor, I provide my opinion on the authors' response to reviewer #1's comments.

As Reviewer #1 pointed out, I agree that a more detailed examination of the Golgi structure with respect to TM9SF3/CCDS22 knockdown would greatly enhance the impact of this paper. The authors claim that the colocalization between cis-Golgi and TGN was decreased by knockdown of TM9SF3 with double immunostaining of a cis-Golgi marker (GM130) and a TGN marker (TGN46) (Figure 4CD). However, I think it would be difficult to quantify the co-localization because the expression level of TGN46 was decreased as shown in Figure 4B. From the photos in Figure 4C, it seems that the fluorescence intensity of TGN has simply decreased, rather than that the cis-Golgi and TGN have dissociated. The authors should describe the detailed method for quantifying the colocalization used for Figure 4D.
Also, I wonder if the cells could be damaged by the transfection procedures, resulting in Golgi fragmentation. the detailed method of KD should be described in the text, including how the control cells were treated.
Another point is that ST and other glycosyltransferases are located in the "trans-Golgi" cisterna of the Golgi apparatus, not in the TGN. The TGN is the compartment specialized for cargo sorting and export, and no glycosylation takes place there. For the purposes of the experiments in this paper, the authors should use a trans-Golgi marker (such as the CTS region of ST or GalT) instead of the TGN marker TGN46.

The analysis of TM9SF3/CCDC22 localization in the Golgi, as pointed out by Reviewer 1, is also very important. Even if specific antibodies are not available, it is possible to use fluorescent protein fusion proteins, even in overexpression systems.

Overall, I feel that the authors have not adequately addressed the points raised by Reviewer #1.

Version 1:

Reviewer comments:

Reviewer #4

(Remarks to the Author)
I think the authors have adequately addressed most of the points raised by myself and the other reviewer, and I applaud the great effort they have made with additional experiments, including CLEM and mass spectrometry. I have only one comment on the authors' rebuttal. Now I understand the reason why the authors used TGN markers (TGN46) instead of trans-Golgi markers in their experiments. However, I would again like to emphasize that the localization of sialyltransferase is in the trans-Golgi, not in the TGN. Although the criteria of trans-Golgi and the TGN are confused and misused by some researchers, the CTS region of sialyltransferases is the best known trans-Golgi marker, and its localization is clearly different from that of the TGN marker TGN46 (e.g., Tie et al, MBoC 2016 DOI: 10.1091/mbc.E15-09-0664), although there is some overlap based on the Golgi cisternal maturation model. I believe that many Golgi researchers share the concept that the trans-Golgi and the TGN should be distinct compartments, although there are some papers where this concept is misunderstood and confusingly stated.
In any case, I would be in favor of accepting the paper in its current form. I trust that this study will have a significant impact on the field of cell biology.

Open Access This Peer Review File is licensed under a Creative Commons Attribution 4.0 International License, which permits use, sharing, adaptation, distribution and reproduction in any medium or format, as long as you give appropriate credit to the original author(s) and the source, provide a link to the Creative Commons license, and indicate if changes were made.
In cases where reviewers are anonymous, credit should be given to 'Anonymous Referee' and the source.
The images or other third party material in this Peer Review File are included in the article's Creative Commons license, unless indicated otherwise in a credit line to the material. If material is not included in the article's Creative Commons license and your intended use is not permitted by statutory regulation or exceeds the permitted use, you will need to obtain permission directly from the copyright holder.
To view a copy of this license, visit https://creativecommons.org/licenses/by/4.0/

Response to reviewer comments:

We thank the reviewers for their insightful comments and suggestions for our manuscript. While the reviewers found our approach compelling and the results interesting, they also expressed a desire for additional mechanistic insights. In response, we have conducted further glycomics and electron microscopy analyses and provided clarifications that we believe significantly enhance the study. Please find a summary of the new data below. . A point-by-point response to individual comments are given below in blue.

Summary of new data in the revised manuscript:

- We performed MALDI-TOF N-analysis on our knockdown cells. The results are in concordance with our lectin microarray data, showing that knocking down TM9SF3 increases high mannose structures while decreasing complex, sialylated bi, tri, and antennary structures. In contrast, knocking down CCDC22 resulted in a shift toward more complex structures and reduced the relative abundance of high mannose structures. (**Extended Data Fig. 9, P.7 and 8**)
- We used correlative light and electron microscopy (CLEM) to further characterize the Golgi changes in our TM9SF3 and CCDC22 knockdown cells (**Extended Data Fig. 7 and 8**). We find fewer intact Golgi stacks in both knockdowns.
- We tested whether knocking down TM9SF3 and CCDC22 in other cell lines would result in similar high mannose changes. We find that knocking down TM9SF3 in K562 and Jurkat cells led to mild to no changes in high mannose levels, whereas knocking down CCDC22 in these other cell lines resulted in a similar reduction in high mannose (**Extended Data Fig. 6**).

Overall, we are confident that these additional experiments, along with the initial findings, represent a powerful approach to understanding glycosylation and provide insights into the regulation of high mannose glycans. We believe this work will be of significant interest to the readers of *Nature Communication*.

Reviewer #2 (Remarks to the Author):

This manuscript focuses on using CRISPR genomic screening to identify genes that regulate presentation of mannose-binding glycans on the cell surface. I previously reviewed this manuscript and thank the authors for their detailed response to my comments. As I said before, the approach is potentially powerful and the authors present some interesting preliminary findings. However, I still feel that the findings of the manuscript are too mechanistically under-developed for publication in its current form. While the authors have assuaged some of my concerns, the majority of my comments have not been sufficiently addressed to change my initial opinion.

Firstly, I still have doubts about the impact and novelty of their findings. To re-state my original comment more precisely, I do not think it is surprising a priori that disrupting a Golgi protein like TM9SF3 would alter cell-surface glycosylation. This has been established in several previous CRISPR screening studies. It would also be intuitively expected given the importance of the Golgi Apparatus in mediating glycosylation in the secretory pathway. My question regarding the biological significance of this finding is largely unaddressed. Is TM9SF3 an essential core structural component of the Golgi machinery that is essential for glycosylation and secretion in all tissues/cell types, like members of the ALG or SEC gene families? Or is it more like a sialyl- or fucosyl-transferase, whose expression/activity is dynamically regulated so as to finely modulate the precise character of protein glycosylation? To me this is the key question to determine how interesting their findings are, and the basic microscopy experiments performed by the authors do not address this point. While they generally comment on the regulation of cell-surface mannose expression in disease, they can't point to any real link between TM9SF3 and these biological functions. This limitation diminishes my enthusiasm for this study. They also have not attempted to study their hit genes in different cell models, which might help address some of these points.

We agree that finding Golgi disruption to impact glycosylation is not surprising. However, one of our more interesting findings is that both TM9SF3 and CCDC22 knockdowns both resulted in Golgi fragmentation but have <u>opposing</u> glycosylation phenotypes. These results would not have been uncovered without our lectin microarray analyses. How these genes precisely alter Golgi and result in drastically different glycosylation phenotypes is an exciting avenue to pursue but beyond the scope of this work.

Our lectin panel results (Extended Data Fig. 3c) show that knocking down TM9SF and CCC complex members shows distinct glycosylation phenotypes compared to COG3 and COG6 knockdowns. In addition, we performed correlative light and electron microscopy (CLEM) to analyze Golgi structures in TM9SF3 and CCDC22 knockdown cells. We do not find Golgi disruption similar to previously reported COG gene deletions (PMID: 31334232), suggesting that they regulate Golgi in a distinct manner as the core Golgi genes. (Extended Data Figures 7 and 8, P. 6 and 7).

We tested whether knocking down CCDC22 or TM9SF3 in two other cell lines, K562 and Jurkat cells, and found that knocking down CCDC22 similarly reduced high mannose levels in both cell lines. On the other hand, knocking down TM9SF3 mildly upregulated high mannose in K562 and downregulated high mannose in Jurkat. These results are now included in Extended Figure 6 and discussed in P.6 and P.7).

Secondly, I have questions regarding the exclusive focus on lectins to characterize glycosylation on their cell line. Lectin arrays are an amazing technology and are useful for many applications, in particular when working with precious samples where the amount of cell material available may be too limited for MS analysis. However, there are advantages to MS-based glycomics in terms of reproducibility and structural resolution. Such techniques can very feasibly be applied to proliferating cell lines and would be extremely helpful in interpreting and validating some of the authors findings. For example, knockout of many of their hits seem to reduce both complex (L-PHA) and high mannose N-linked glycosylation, which is somewhat counter-intuitive to me. A global MS profiling dataset of at least some gene knockouts would help clear up exactly what the impact of gene knockout is on glycosylation. Mass Spec glycomics analysis is available as a service through several NIH-funded core facilities, and I don't think it's unreasonable to expect some of this characterization when working with cultured cells.

We performed N-glycan analysis on TM9SF3 and CCDC22 knockdown cells, and the results are in concordance with our lectin microarray results (Extended Data Fig. 9, P. 7 and 8). We see a clear shift towards high mannose structures in TM9SF3 knockdown cells and complex structures in CCDC22 knockdown cells. In CCDC22 knockdown cells, we also see an increase in sialylated and LacNAc structures, similar to the changes we observed with our lectin microarrays.

To clarify, we do not see or report a reduction in high mannose and complex glycans (L-PHA) in our results. We see slight upregulation of complex glycans (L-PHA) and down-regulation of high mannose in our lectin analysis of CCDC22-KD (Fig. 5e and f). The glycomics analysis shows the same result – with decreased high mannose and slight upregulation of tri- and tetra-antennary structures (Extended Data Fig. 9). Similarly, in our lectin staining panel in Extended Data Fig. 3c, we see a downregulation in high mannose GRFT staining, but no significant change in PHA-L staining, which might be because the upregulation is too small to be detected and/or that changes on the cell surface is minimal compared to whole cell glycans. We have updated the color scheme of the heatmap to make this clearer.

Reviewer #3 (Remarks to the Author):

The authors have adequately addressed most of the questions. I will look forward to seeing the manuscript in print.

Reviewer #4 (Remarks to the Author):

Regarding the paper submitted to Nat Comm by Tsui et al, at the request of the editor, I provide my opinion on the authors' response to reviewer #1's comments.

As Reviewer #1 pointed out, I agree that a more detailed examination of the Golgi structure with respect to TM9SF3/CCDS22 knockdown would greatly enhance the impact of this paper. The authors claim that the colocalization between cis-Golgi and TGN was decreased by knockdown of TM9SF3 with double immunostaining of a cis-Golgi marker (GM130) and a TGN marker (TGN46) (Figure 4CD). However, I think it would be difficult to quantify the co-localization because the expression level of TGN46 was decreased as shown in Figure 4B. From the photos in Figure 4C, it seems that the fluorescence intensity of TGN has simply decreased, rather than that the cis-Golgi and TGN have dissociated. The authors should describe the detailed method for quantifying the colocalization used for Figure 4D.

In the TM9SF3 knockdowns, we agree that the decreased co-localization is partly due to reduction in TGN and have stated so in the text. We think that both the cis-Golgi fragmentation as well as the decrease in TGN leads to an imbalance of cis- and trans-Golgi, corresponding to an increase of unpaired cis-Golgi. This, we believe, leads to the upregulation of under-processed, high mannose glycans on the cell surface. We have rewritten parts of the text to clarify this model (P.6). We have also included additional details of the quantification method in the figure legends.

Furthermore, to gain insight into Golgi disruptions, we used correlative light and electron microscopy (CLEM) to analyze Golgi structures in TM9SF3 and CCDC22 knockdown cells. We find knocking down TM9SF3 led to less elaborate Golgi stacks (Extended Data Figure 7, P. 6). Interestingly, knocking down CCDC22 led to an accumulation of unknown vesicular structures (Extended Data Figure 8, P.7).

Also, I wonder if the cells could be damaged by the transfection procedures, resulting in Golgi fragmentation. the detailed method of KD should be described in the text, including how the control cells were treated.

None of the knockdowns are done using transfection but stable knockdowns using lentiviral methods, so there would not be any artifacts from transfection procedures. We have clarified this in the methods (P.10)

Another point is that ST and other glycosyltransferases are located in the “trans-Golgi” cisterna of the Golgi apparatus, not in the TGN. The TGN is the compartment specialized for cargo sorting and export, and no glycosylation takes place there. For the purposes of the experiments in this paper, the authors should use a trans-Golgi marker (such as the CTS region of ST or GalT) instead of the TGN marker TGN46.

Sialyltransferases and galactotransferases (PMID: 3095318, 3000603,19261593) are present in the TGN, and the TGN has been shown to be a site of sialylation (PMID: 30659093,33524390). We have tried several antibodies targeting glycosyltransferases that are typically localized to the trans-Golgi but could not find one that worked in our hands. We also do not want to overexpress a trans-Golgi marker (such as fluorescent protein fused to the CTS region of ST or GalT) as that has been associated with altered localization. We believe that the well-characterized TGN46 marker is sufficient for our characterization. We have gone back to the text to clarify that we are assaying the trans-Golgi network but not the trans-Golgi.

The analysis of TM9SF3/CCDC22 localization in the Golgi, as pointed out by Reviewer 1, is also very important. Even if specific antibodies are not available, it is possible to use fluorescent protein fusion proteins, even in overexpression systems.

As referenced in the text, TM9SF3 has been previously reported to be localized to the Golgi, and CCDC22, along with the CCC complex, is enriched in the endosome and cytoplasm. Given that these have been shown multiple times in the literature, we do not think repeating this will add much to the study.
